# *DEK* Loss Induces Task-specific Deficits in Learning and Memory and Reprograms the Hippocampal Transcriptome in Mice

**DOI:** 10.1007/s12035-026-06022-4

**Published:** 2026-06-29

**Authors:** Kaitlyn L. Gardner, Taylor E. Lange, Marla K. Perna, Susanne I. Wells, Michael T. Williams, Charles V. Vorhees, Marley Cox, Matia B. Solomon, Lisa M. Privette Vinnedge

**Affiliations:** 1 Division of Oncology, Cincinnati Children’s Hospital Medical Center, Cincinnati, OH USA; 2 Department of Cancer Biology, University of Cincinnati College of Medicine, Cincinnati, OH USA; 3 Division of Neurology, Cincinnati Children’s Hospital Medical Center, Cincinnati, OH USA; 4 Department of Pediatrics, University of Cincinnati College of Medicine, Cincinnati, OH USA; 5 Department of Psychology, University of Cincinnati College of Arts and Sciences, Cincinnati, OH USA

**Keywords:** *DEK*, Cognitive dysfunction, Sex differences, Memory, Hippocampus, Morris Water Maze, Cognitive flexibility, Reversal learning, Chromatin

## Abstract

DEK is an estrogen-responsive chromatin-remodeling protein broadly expressed in the murine and human brain, with high expression in memory-relevant regions such as the hippocampus. Prior work from our group and others has linked DEK loss to cellular features associated with Alzheimer’s disease and Alzheimer’s disease-related dementias. Notably, our group has demonstrated that DEK expression declines with increasing dementia severity in women, but not in age-matched men, suggesting a sex-specific relationship between DEK loss and cognitive vulnerability. Together, these findings support a potential neuroprotective role for DEK; however, functional consequences of DEK loss in vivo were unknown. Here, we examined behavioral and molecular consequences of *Dek* loss using male and female constitutive knockout (cKO) mice assessed across cognitive, sensorimotor, and affective domains. Across assays, *Dek* cKO mice of both sexes exhibited intact locomotor activity, anxiety-related behavior, sensorimotor gating, and fear-associated memory. In contrast, female *Dek* cKO mice displayed selective impairments in cognitive flexibility despite preserved spatial learning and memory, a phenotype not observed in males and indicative of female-specific vulnerability following DEK loss. This sex difference, observed during Morris water maze reversal learning, suggests disruption of hippocampal–prefrontal circuitry. Guided by known sex differences in hippocampal DEK expression, transcriptomic profiling of hippocampal tissue revealed shared and sex-specific consequences of *Dek* deficiency, including alterations in cytoskeletal organization, neuronal signaling, chromatin regulatory mechanisms, and cellular stress pathways. Collectively, these findings demonstrate sex- and cognitive-domain-specific effects of DEK loss and support further investigation of DEK in executive function and hippocampal–prefrontal cortex–mediated cognition.

**Supplementary Information:**

The online version contains supplementary material available at 10.1007/s12035-026-06022-4.

## Introduction

Cognitive functions such as learning, memory, and cognitive flexibility depend on tightly regulated biological processes within the brain. Accumulating evidence indicates that regulation of chromatin structure and gene expression plays a fundamental role in supporting these cognitive processes, particularly by enabling experience-dependent and long-lasting changes in neural function [1, 2]. Disruption of chromatin-based regulatory mechanisms has been associated with selective alterations in cognitive performance [3, 4]. These mechanisms may also operate differently across biological contexts, including sex, contributing to variability in cognitive function.

One factor that may contribute to sex-specific differences in cognitive function is the *DEK* protein. *DEK*, an estrogen receptor alpha (ER-α) target gene, is a chromatin remodeling protein essential for DNA replication, genome stability, chromatin organization, and transcriptional regulation [5–7]. *DEK* is expressed in almost all tissues including the brain, but primarily in proliferating progenitor cells [8, 9]. Most previous reports have focused on the role of DEK in solid tumors, where it is commonly overexpressed [10–16]. Our group and others have shown that *DEK* loss in cancer cell lines leads to impaired activation of DNA damage repair pathways [17, 18], cellular senescence [19], and cell death [20–23]. In addition, utilizing differentiated SH-Sy5y cells, we demonstrated that *DEK* loss correlated with cellular and molecular phenotypes of Alzheimer’s disease (AD) including cell death and increased pathogenic Tau accumulation [24], which was supported by in vitro and in vivo findings [24–26]. We were the first to report that *Dek* is expressed in several brain regions that are important to learning and memory (e.g., hippocampus, medial prefrontal cortex), and that it is likely expressed in many cell types including astrocytes, neurons, and microglia in both the mouse and human brain [9, 27]. Furthermore, we reported sex-specific differences in DEK expression in the murine brain, including the finding that females have higher DEK protein expression in the CA1 region of the hippocampus when compared with males [9]. Using human postmortem brain tissue, we determined that older women with schizophrenia-associated dementia have decreased DEK expression in the anterior cingulate cortex, a region important for integration of emotional and cognitive information, compared with their healthy sex- and age-matched controls, with an inverse correlation that indicated decreasing DEK levels with increasing dementia severity [28]. Notably, this association was not evident in elderly men with schizophrenia-associated dementia, suggesting a female-biased link with DEK loss and dementia. Using transcriptomic data, we also showed that *DEK* levels are highest in the fetal human brain and decrease with age [27]. *DEK* was also identified as a differentially expressed gene in humans with neurodegenerative Huntington’s disease [29]. Combined, work from our group and others indicates that *DEK* loss is associated with molecular and cellular correlates of cognitive dysfunction and may function in a sex-specific manner.

This study aims to bridge the gap between DEK protein expression data from murine and human brains by examining the sex-specific functional and molecular consequences of DEK loss on memory in adult female and male *Dek* constitutive knockout (cKO) mice compared with wild-type (WT) control mice. Given our previous findings that lower DEK protein levels are associated with greater dementia severity in elderly women but not elderly men [28], we hypothesized that female *Dek* cKO mice would exhibit behavioral phenotypes indicative of cognitive impairment, cognitive inflexibility, or behavioral dysfunction. To examine the molecular consequences of *Dek* loss in the brain using bulk RNA-seq, we focused on the hippocampus because it exhibits the highest density of DEK-positive cells in the brain (particularly in the dentate gyrus) [9], shows reported sex differences in DEK expression in the CA1 region [9], plays an essential role in memory, and is prominently affected in neurodegenerative disorders associated with memory impairment, such as AD. Here, we show that *Dek* loss results in task- and sex-dependent cognitive effects accompanied by a distinct hippocampal transcriptomic signature. These changes include shared alterations across sexes, such as genes associated with pro-inflammatory signaling and neuronal structural plasticity, as well as sex-specific transcriptomic signatures for genes related to cellular response to proteotoxic stress, chromatin remodeling, and Wnt signaling.

## Results

### Dek Loss Leads to Task-specific Cognitive Impairments that Differs Between the Sexes

**Fig. 1:**
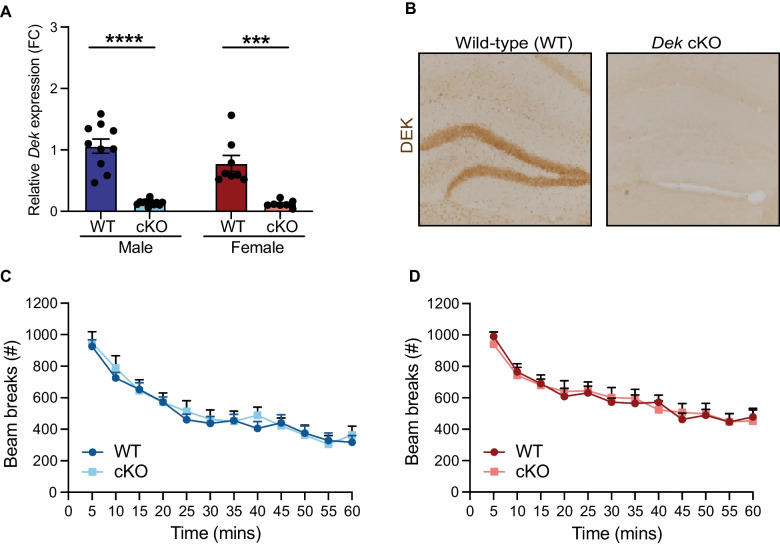
Loss of DEK expression does not impair overall locomotor activity in the open field in adult mice. **A** qRT-PCR analysis of relative *Dek* mRNA expression (fold change, FC) confirms a significant reduction in *Dek* expression in cKO compared with wild-type (WT) mice (**B**) Representative immunohistochemical staining showing DEK expression in hippocampal sections from wild-type (WT) and *Dek* conditional knockout (cKO) mice, demonstrating markedly reduced DEK protein expression. **C**, **D** Beam-break activity over time during the open field in male (**C**) and female (**D**) mice. Both WT and cKO animals show comparable locomotor activity over the one-hour testing session. Dark and light blue bars represent males, and red and pink bars represent females. All mice were adults. For qRT-PCR: *N* = 8 WT females, 8 *Dek* cKO females, 10 WT males, 11 *Dek* cKO males. For open field: *N* = 12 WT females, 10 *Dek* cKO females, 22 WT males, 14 *Dek* cKO males. Data are shown as mean ± SEM with individual data points; **p < 0.001, ***p < 0.0001

To investigate the effect of *Dek* loss on behavior, *Dek* cKO mice were generated by breeding the *Dek*^*fl/f*l^ mice with CMV-Cre mice to generate a constitutive knockout (cKO) as reported in [30]. Loss of *Dek* expression was confirmed in *Dek* constitutive knockout (cKO) mice using qRT-PCR. *Dek* mRNA levels were significantly reduced in cKO mice compared with same-sex wild-type (WT) controls in both males, *t*(9.20) = 6.68, *p* < 0.0001, and females, *t*(7.43) = 5.98, *p* < 0.0001 (Fig. 1A). Immunohistochemical analysis further demonstrated robust DEK protein expression in the dentate gyrus of the hippocampus in WT mice, whereas DEK immunoreactivity was absent in *Dek* cKO mice (Fig. 1B). A similar loss of DEK protein expression was observed throughout the brain. The marked reduction of DEK expression in cKO mice confirms successful gene deletion and supports the use of this model to evaluate the functional role of DEK in learning and memory.

Open-field activity was assessed for 1 h to ensure that there was no impact of *Dek* loss on locomotor activity including exploration or habituation activity. Locomotor activity, indexed by total beam breaks in the open field, was analyzed using a linear mixed-effects model with genotype (WT vs. *Dek* cKO) as a between-subjects factor and time as a repeated measure. There was a robust main effect of time in both males (*F*(11, 321) = 29.30, *p* < 0.0001; Fig. 1C) and females (*F*(11, 187) = 27.23, *p* < 0.0001; Fig. 1D), reflecting higher activity early in the session followed by a progressive decline over time which is consistent with normal open-field habituation. In contrast, there was no main effect of genotype and no genotype × time interaction in either males or females (both *p* > 0.05), indicating comparable overall locomotor activity and similar habituation profiles across genotypes. Notably, *Dek cKO* mice did not differ from WT mice of either sex in their preference for central versus peripheral movement, indicating no genotype-related differences in anxiety-like behavior (data not shown).

**Fig. 2:**
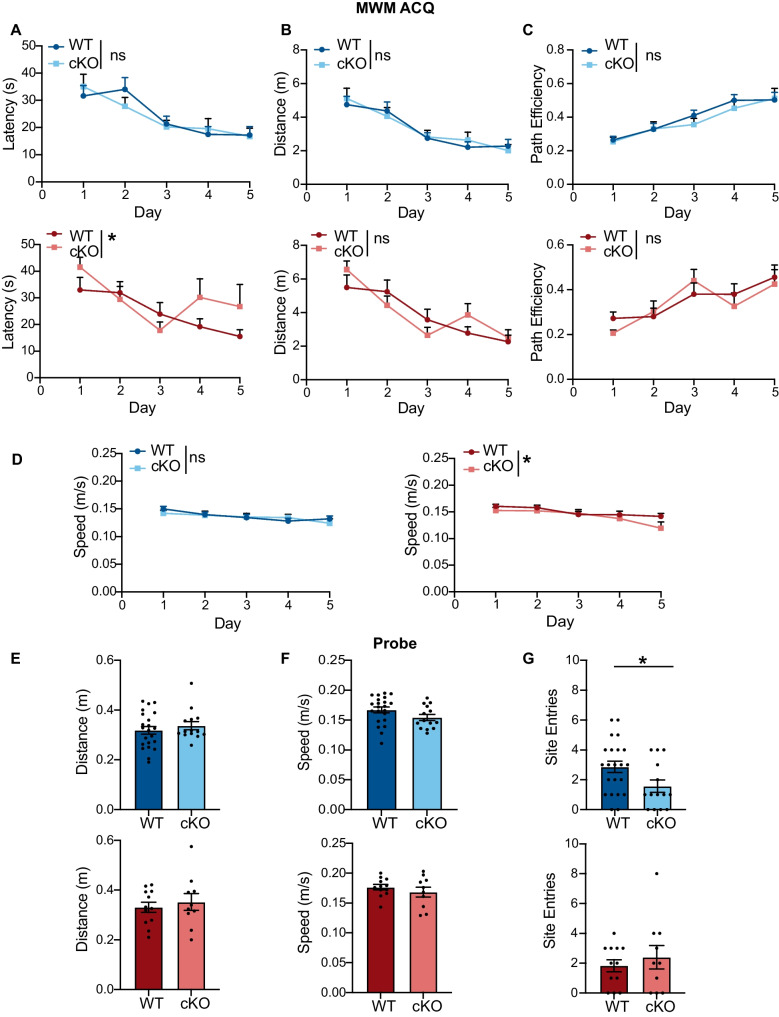
Spatial learning and reference memory are largely preserved in male and female *Dek* cKO mice. **A**–**D** Acquisition. Morris water maze acquisition performance shown as escape latency (**A**), swim distance (**B**), path efficiency (**C**) and swim speed (**D**) across 5 days of training, in males (top panels, blue) and females (bottom panels, pink/red). Male WT and *Dek* cKO mice exhibited comparable reductions in latency and distance across training, with no genotype differences in path efficiency or swim speed, indicating intact spatial learning and motor performance. Female *Dek* cKO mice showed modest increases in escape latency on later training days relative to WT females (days 4 and 5), while distance traveled improved similarly across training in both genotypes with no differences in path efficiency. Swim speed was largely comparable between female groups across training, with a modest reduction observed in *Dek* cKO females on the final day.** E**–**G** Probe trial. Performance during the probe trial, shown as average distance from the former platform location (**E**), swim speed (**F**), and platform site entries (**G**), in males (top panels) and females (bottom panels). Male *Dek* cKO mice made fewer platform site entries than WT males, whereas no genotype differences were observed for average distance from the platform location or swim speed. Female WT and *Dek* cKO mice did not differ in any probe measures. Adult mice were used for the behavioral analyses: *N* = 12 WT females, 10 *Dek* cKO females, 22 WT males, 14 *Dek* cKO males. Data are presented as mean ± SEM with individual data points shown. WT, wild-type; cKO, Dek conditional knockout; ns, not significant; *p < 0.05

We first performed the novel object recognition (NOR) assay, according to previously published methods from our group [31] and found no significant differences using a four-object experimental design (data not shown). To explore the effects of *Dek* cKO loss on spatial learning, reference memory, and cognitive flexibility, the Morris water maze (MWM) was used. Before assessing spatial learning, mice were given cued training in the maze from a fixed start to a fixed and visible platform. There were no significant genotype effects on training trials in either males or females (not shown). Next, mice were tested for spatial learning to find a hidden platform with different start positions on each trial with 4 trials/day for 5 days. For males, there were no differences in latency (Fig. 2A top panel), distance (Fig. 2B top panel), path efficiency (Fig. 2C top panel) or swimming speed (Fig. 2D left panel). These findings indicate that male *Dek* cKO mice performed comparably to WT male controls across multiple measures of performance.

In contrast, in females there was a significant interaction of genotype × day for latency (*F*(4,52.7) = 2.87, *p* = 0.0319, Fig. 2A bottom panel); for which the Kenward-Roger first-order adjusted degrees of freedom were calculated for Type III ANOVAs [32]. With this method, degrees of freedom can be fractional. Further analyses showed this effect trends were on later days (days 4–5) wherein female *Dek* cKO mice had higher latencies than WT females (*p*’s = 0.10). There were no differences between females for distance (Fig. 2B bottom panel) or path efficiency (Fig. 2C bottom panel). Path efficiency improved over time for both groups; *F*(4, 47.4) = 9.10, *p* < 0.0001 suggesting normal spatial learning during acquisition (Fig. 2C). There was a trend for a genotype × day interaction for swimming speed in females (*F*(4,57.1) = 2.39, *p* = 0.0617; Fig. 2D right panel). Further analyses using the slice option in Proc Mixed revealed a significant genotype difference on day 5 (*F*(1, 38.04) = 5.45, *p* = 0.0249), with *Dek* cKO females exhibiting slower swim speeds than WT females. Together, these data indicate that female *Dek* cKO mice exhibit intact spatial learning and navigational strategy during acquisition. Although female *Dek* cKO mice showed a modest reduction in swim speed on the final day of acquisition, swim speed was otherwise comparable across training.

Twenty-four hours after the last acquisition trial, mice were given a probe trial with the platform removed as a test of reference memory. For the males, there were no differences in the average distance from the platform site or swim speed (Fig. 2E, F, top panels). However, male *Dek* cKO mice made significantly fewer platform site entries than WT males (*t*(34) = 2.23, *p* = 0.033; Fig. 2G, top panel). There were no differences between the females for average distance from the platform site, site entries or speed during the probe trial (Fig 2E–G, bottom panels). Overall, the probe trial performance suggests intact reference memory in both sexes, with only a modest reduction in platform-directed search behavior (site entries) in male *Dek* cKO mice.

**Fig. 3:**
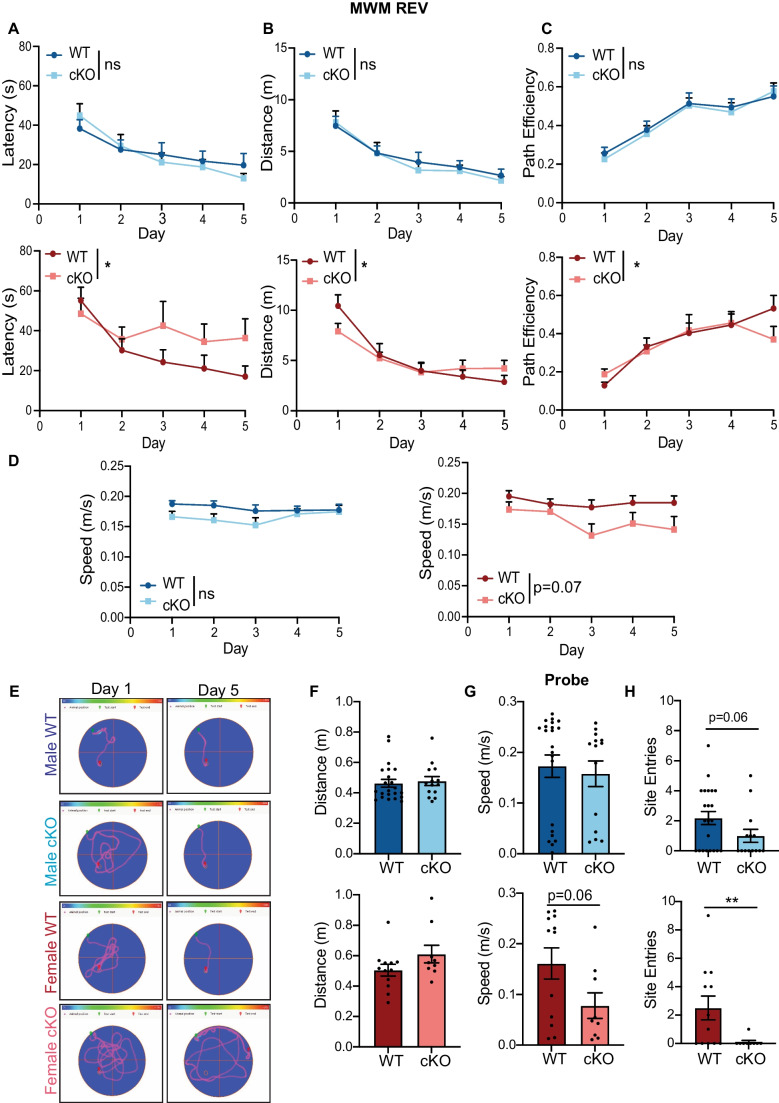
Sex-specific impact of DEK loss on cognitive flexibility during Morris water maze reversal learning. **A**–**C** Reversal. Morris water maze reversal acquisition (MWM REV) performance in male and female *Dek* cKO mice. Reversal learning performance is shown as escape latency (**A**), swim distance (**B**), path efficiency (**C**) and swim speed (**D**) across 5 days of reversal acquisition training in males (top panels, blue) and females (bottom panels, pink/red). Male WT and *Dek* cKO mice exhibited comparable reductions in escape latency and swim distance across training, with no genotype differences in swim speed, indicating intact cognitive flexibility and motor performance. In contrast, female *Dek* cKO mice showed impaired reversal performance relative to WT females, reflected by increased escape latencies on some training days (days 4 and 5). There was a strong trend for a genotype × day interaction (*p* = 0.052) for distance. Female WT mice traveled further on day 1 of reversal acquisition, whereas female *Dek* cKO mice traveled greater distances on the final day. Swim speed in females was also reduced in *Dek* cKO mice during later reversal training, whereas no differences were observed during early acquisition, indicating that slower navigation emerged selectively during later stages of reversal learning. Together, these patterns suggest impaired updating of spatial behavior during reversal acquisition (cognitive inflexibility) in female *Dek* cKO mice rather than overall deficits in initial learning or gross motor ability. **E** Representative swim paths from day 1 and day 5 of reversal training for male WT, male *Dek* cKO, female WT, and female *Dek* cKO mice illustrate efficient updating of search strategies in both male groups and WT females, with less efficient platform-directed navigation evident in female *Dek* cKO mice. The platform location is marked with an open circle, the pink indicates the swimming path of the mouse, the green dot marks the starting location, and the red dot marks the ending location. **F**–**H** Probe. Performance during the probe trial is reported as platform site entries (**F**), average distance from the former platform location (**G**), and swim speed (**H**), plotted separately for males (top panels) and females (bottom panels). Male WT and *Dek* cKO mice did not differ across probe measures. In contrast, female *Dek* cKO mice made significantly fewer platform site entries compared with WT females, despite comparable swim speed and average distance, further supporting a female-specific impairment in reversal learning and cognitive flexibility. Adult mice were used for the behavioral analyses: *N* = 12 WT females, 10 *Dek* cKO females, 22 WT males, 14 *Dek* cKO males. Data are presented as mean ± SEM with individual data points shown. WT, wild-type; cKO, Dek conditional knockout; ns, not significant; *p < 0.05; **p < 0.01; trend-level effects are noted where applicable

Cognitive flexibility was assessed during the MWM reversal phase. In males, there were no genotype differences across any measures including latency, distance, path efficiency, or speed (Fig. 3A–C top panel, Fig. 3D left panel). Path efficiency increased progressively across reversal training *F*(4, 103) = 22.40, *p* < 0.0001, indicating progressive navigational efficiency and adaptation to the new platform location (Fig. 3C, top panel). Together these data indicate intact strategy adaptation and preserved cognitive flexibility during reversal learning in male *Dek* cKO mice.

For females there was a genotype × day interaction on latency during reversal (*F*(4,61.2) = 3.54, *p* = 0.0115) (Fig. 3A, bottom panel**)**. Further analyses of each day showed a trend on day 3 (*p* = 0.096) and a significant effect on day 5 (*p* = 0.0220) with female *Dek* cKO mice having longer latencies during reversal learning. A genotype × day interaction was also observed (*F*(4, 61.6) = 2.49, *p* = 0.0525) on distance. Further analyses revealed that on reversal day 1, female *Dek* cKO mice traveled less distance than WT controls, whereas on later reversal days (days 4–5), female *Dek* cKO mice traveled greater distances than WT females. On the final day of reversal, *Dek* cKO females had longer path lengths than WT controls, despite comparable early trial performance (Fig. 3B, bottom panel). The increased path length on later reversal days suggests difficulty optimizing or refining the new strategy, rather than impaired spatial navigation in female *Dek* cKO mice. For path efficiency there was a genotype × day effect (*F*(4,61.5) = 2.57, *p* = 0.0469) in which *Dek* cKO females exhibited less efficient paths to the goal than WT females on day 5 (*p* = 0.0243) (Fig. 3C, bottom panel). For swimming speed, there was a genotype trend (*F*(1,20.3) = 3.46, *p* = 0.0776) in which WT females swam faster than *Dek* cKO females (WT: 0.18 m/s vs cKO: 0.15 m/s; Fig. 3D, right panel). However, there was a significant genotype × day interaction (*F*(4, 63.1) = 2.75, *p* = 0.0358). Post hoc analyses revealed no genotype differences during reversal training on days 1–2. In contrast, female *Dek* cKO mice swam slower than WT controls on day 3 (*F*(1,29.35) = 5.86, *p* = 0.0219) and day 5 (*F*(1,30.25) = 6.11, *p* = 0.0193), with a trend on day 4. Representative swim path tracings illustrate this difference in navigational efficiency (Fig. 3E). Together, these findings indicate that female *Dek* cKO mice retain spatial memory at the beginning of reversal testing. In contrast, the emergence of increased path length, reduced path efficiency, increased latency, and the late-phase slower speed indicate impaired adaptation and hence reduced cognitive flexibility.

On the reversal probe trial, in males, there were no differences in average distance to the platform site (Fig. 3F, top panel) or swimming speed (Fig. 3G, top panel). There was a genotype trend for site entries (*t*(32.36) = − 1.92, *p* = 0.06; Fig. 3H, top panel). Male *Dek* cKO mice had fewer site entries than WT males. Overall, probe-trial performance in males indicates preserved spatial memory in *Dek* cKO mice as shown by similar proximity (distance) to the former platform location. However, there is a subtle reduction in focused, goal-directed search, as evidenced by fewer platform site entries, suggesting a change in search strategy or persistence at the target location. In females, there were no differences between genotypes for average distance to the platform site (Fig. 3F, bottom panel); however, swimming speed showed a genotype trend (*t*(19) = − 1.99, *p* < 0.0609; Fig. 3G, bottom panel); female *Dek* cKO mice had lower swim speeds than WT females. There was a significant genotype effect (*t*(11.384) =  − 2.82, *p* < 0.0161) in which *Dek* cKO females had fewer site entries than WT females (Fig. 3H, bottom panel). Together, probe-trial performance indicates that female *Dek* cKO mice remember the platform location, as evidenced by preserved proximity to the platform site, but less focused searching to the exact target site. This pattern may reflect differences in search strategy, rather than impaired spatial memory.

A day after the reversal probe trial, mice were given cued/visible platform trials as a test of proximal cue learning for 4 trials/day for 2 days with curtains closed around the pool, a marked platform and with both start and platform positions changed on every trial. There were no genotype effects on latency or speed during these trials for males or females (Supplementary Figs. S1A–D). Equivalent performance across genotypes in the cued/visible platform task during the reversal phase indicates that *DEK* loss did not impact visual function, motor ability, or the use of proximal cues to guide behavior. Notably, this was consistent with the absence of genotype differences during cued training in the acquisition phase, indicating that sensorimotor and perceptual processes were intact throughout testing. Together, these findings support the interpretation that deficits in female *Dek* cKO mice observed in the reversal phase of the learning Morris water maze reflect altered cognitive flexibility rather than learning, sensory or motor impairments.

**Fig. 4:**
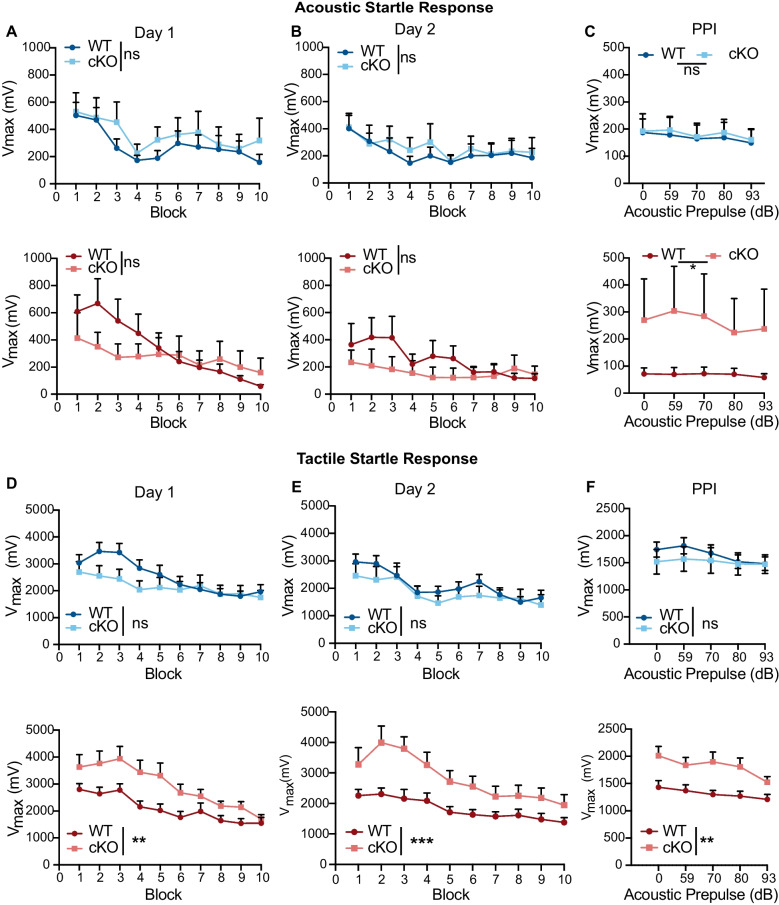
*Dek* loss results in sex-specific impairment in tactile startle response but not acoustic startle responses. **A**–**C** Acoustic Startle Response (ASR). Acoustic startle magnitude (Vmax) across repeated stimulus blocks on day 1 (**A**) and Day 2 (**B**), and acoustic prepulse inhibition (PPI; **C**), in males (top panels, blue) and females (bottom panels, pink/red). In males, WT and *Dek* cKO mice exhibited comparable acoustic startle responses and habituation across sessions, as well as intact ASR-PPI, indicating preserved baseline acoustic startle reactivity and sensorimotor gating. In females, *Dek* cKO mice did not display robust or sustained alterations in baseline acoustic startle magnitude or habituation and failed to identify a particular prepulse level associated with this interaction. **D**–**F** Tactile Startle Response (TSR). Tactile startle magnitude (Vmax) across repeated stimulus blocks on day 1 (**D**) and Day 2 (**E**), and tactile PPI (**F**), in males (top panels, blue) and females (bottom panels, pink/red). Male WT and *Dek* cKO mice exhibited comparable tactile startle magnitudes and intact tactile PPI across sessions, indicating preserved tactile responsivity and sensorimotor gating. In contrast, female *Dek* cKO mice showed significantly increased tactile startle magnitudes relative to WT females across both testing days, accompanied by increased tactile PPI. This pattern indicates heightened tactile responsivity in female *Dek* cKO mice with preserved, rather than impaired, prepulse-mediated inhibition, while basic acoustic startle reactivity remains largely intact. Adult mice were used for the behavioral analyses: *N* = 12 WT females, 10 *Dek* cKO females, 22 WT males, 14 *Dek* cKO males. Data are presented as mean ± SEM. Vmax, maximum startle amplitude. ns, not significant; Statistical significance is indicated as *p < 0.05, **p < 0.01, ***p < 0.001; trend-level effects are noted where applicable

*Dek* cKO mice were assessed for acoustic and tactile startle responses to examine sensorimotor responsiveness. For the acoustic startle response (ASR), there was no effect of genotype on either testing day (days 1 and 2) in males or females (Fig. 4A, B, top and bottom panels), indicating that *Dek* loss does not significantly impact baseline acoustic startle reactivity.

Tactile startle response (TSR) revealed a sex-specific effect. There were no genotype differences in TSR in males on either day 1 or day 2 (Fig. 4D, E, top panels). However, female *Dek* cKO mice exhibited significantly increased tactile startle responses compared with WT females on both day 1 (*F*(1,22.7) = 10.48, *p* = 0.0037; Fig. 4D, bottom panel) and day 2 (*F*(1,21.4) = 17.19, *p* = 0.0004; Fig. 4E bottom panel) of testing, indicating enhanced tactile reactivity in females with *Dek* loss.

On day 3, mice were given prepulse inhibition trials (PPI) with an acoustic prepulse and an acoustic or tactile pulse. There were no effects for acoustic (Fig. 4C, top panel) or tactile PPI (Fig. 4F, top panel) in males. For acoustic PPI in females there was no genotype effect but there was a genotype × prepulse interaction (*F*(4,54) = 2.50, *p* = 0.0528) (Fig. 4C, bottom panel). Further analyses at each prepulse level failed to identify a particular prepulse level associated with this interaction. On trials with a tactile pulse, for females there was a genotype main effect (*F*(1,20.6) = 10.69, *p* = 0.0037) (Fig. 4F, bottom panel). In both cases of PPI testing, *Dek* cKO females were hyperreactive compared with WT females.

Contextual and cued freezing conditioning were used to determine the necessity of *Dek* expression in fear-related learning and memory. The task distinguishes memory for an aversive context from memory for a specific predictive cue, allowing detection of selective alterations in fear memory expression. Both male and female WT and *Dek* cKO mice exhibited comparable levels of freezing during initial context exposure on day 1 (habituation), indicating no effects of genotype on baseline freezing behavior (Supplementary Fig. S2A, B). During conditioning on day 2, pre-conditioning activity was minimal in all groups, while US–UR pairings elicited increases in freezing in both WT and *Dek* cKO mice of both sexes, with no genotype-related differences observed, demonstrating intact acquisition of conditioned fear in males and females (Supplementary Figs. S2C, D).

Contextual memory was assessed on day 3. In males, *Dek* cKO mice exhibited less freezing than WT mice; however, this difference did not reach statistical significance but showed a trend toward reduced contextual memory in cKO males (*p* = 0.057; Supplementary Fig. S2E). In contrast, female WT and cKO mice displayed similar levels of contextual freezing (Supplementary Fig. S2F).

When cued memory was assessed on day 4 in a novel environment, freezing during the stimulus-free interval and during light presentation was comparable between WT and cKO mice in both males and females, with no genotype-dependent differences detected in either sex (Supplementary Fig. S2G, H).

Extinction was then evaluated across blocks on day 4. During tone presentation, WT and cKO mice of both sexes exhibited similar freezing responses and parallel trajectories across blocks, indicating comparable within-session movement and no evidence for genotype × block interactions in males or females (Supplementary Fig. S2I, J). Freezing during tone-off periods likewise did not differ between genotypes in either sex (Supplementary Fig. S2K, L).

Together, the data indicate that fear learning and memory are intact in both male and female *Dek* cKO mice, as evidenced by normal acquisition of conditioned freezing during training and preserved expression of both contextual and cued freezing during memory testing. Additionally, normal within-session modulation of freezing across testing blocks supports intact regulation of previously learned freezing responses.

### Sex Differences in the Transcriptomic Consequences of DEK Loss

**Fig. 5:**
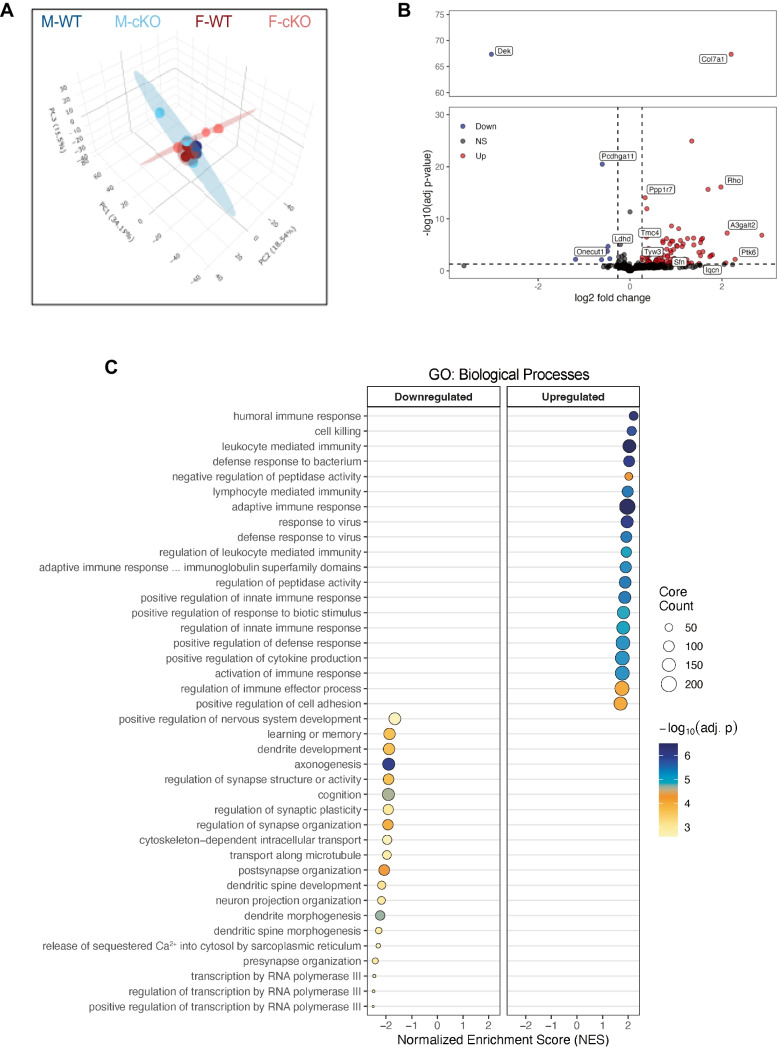
The hippocampus of *Dek* knockout mice has a unique transcriptomic profile characterized by differential expression of genes linked to dendrite morphogenesis, inflammation, and immune responses. **A** Principal component analysis (PCA) shows the separation of groups based on sex and genotype due to variation in the transcriptome using hippocampal mRNA. The ellipses represent 95% confidence intervals. N = 3/genotype/sex. **B** The volcano plot depicts genes upregulated (red) and downregulated (blue) in *Dek* cKO hippocampus compared with WT mice. **C** Gene Set Enrichment Analysis for Biological Processes using DeSeq2-identified differential gene expression, shows up-regulation of immune response and inflammatory gene sets and down-regulation of gene sets linked to dendrite morphogenesis, synaptic plasticity, and regulation of transcription. Dot size represents the number of genes in the gene set differentially expressed in *Dek* cKO tissue vs WT tissue. The color scale represents *p* value, with light yellow indicating lower *p *values. The *X* axis graphs the gene set enrichment based on Normalized Enrichment Score (NES). Negative NES values represent down-regulation of a gene set and positive NES values depict up-regulation of a gene set

Given our prior evidence of sex-specific DEK expression that is unique to the hippocampus [9], we focused our transcriptomic analyses on this region due to its central role in long-term and spatial memory, and its association with performance in the MWM. We performed bulk RNA-seq on micro-dissected hippocampal tissue from male and female wild-type and *Dek* cKO mice as an exploratory, hypothesis-generating approach to identify shared and sex-specific molecular consequences of *Dek* loss in a learning and memory-relevant brain region. Quality control information for the samples used for RNA-seq is shown in Supplementary Fig. S3. Three-dimensional principal component analysis (PCA) confirmed the clustering of samples within each group. Minimal difference in gene expression between the sexes of WT mice was confirmed by the overlap in male and female clusters (Fig. 5A) and the similarity in transcriptomic profiles as shown in a heatmap (Supplementary Fig. S4). In contrast, PCA of male and female *Dek* cKO mice displayed greater separation from each other, supporting greater variability between samples, and separation from their respective WT groups. We first compared *Dek* cKO mice with WT mice, regardless of sex, using DeSeq2 to identify differentially expressed genes (DEGs). There were 113 DEGs, with 95 genes upregulated, and only 18 genes downregulated, between WT and *Dek* cKO mice (adj *p* value < 0.05, ± 1.2-fold change; Fig. 5B). We also assessed the number of DEGs with significant raw *p* values since gene set enrichment analysis (GSEA) utilizes a ranked list of all expressed genes in a dataset, including nominally changed genes, to identify statistically significant pathways. When considering raw/nominal *p* values < 0.05 and fold change ± 1.5, there were 428 upregulated genes and only 80 down-regulated genes. This skewing was expected, given the role of *DEK* in promoting heterochromatin formation for gene silencing [33–36]. We performed gene set enrichment analysis (GSEA) and found that the hippocampal transcriptomes of *Dek* cKO mice were enriched for changes in biological processes necessary for brain health, dendrite formation, and neuron function. Specifically, *Dek* cKO hippocampal transcriptomes indicated repression of processes associated with RNA polymerase III-mediated transcription, synapse organization, dendritic spines and dendrite morphogenesis, learning/memory and cognition, and transport along microtubules. However, the upregulated biological processes in *Dek* cKO hippocampi were overwhelmingly associated with adaptive and innate immune responses, suggesting neuroinflammation, along with regulation of peptidase activity (Fig. 5C).

**Fig. 6:**
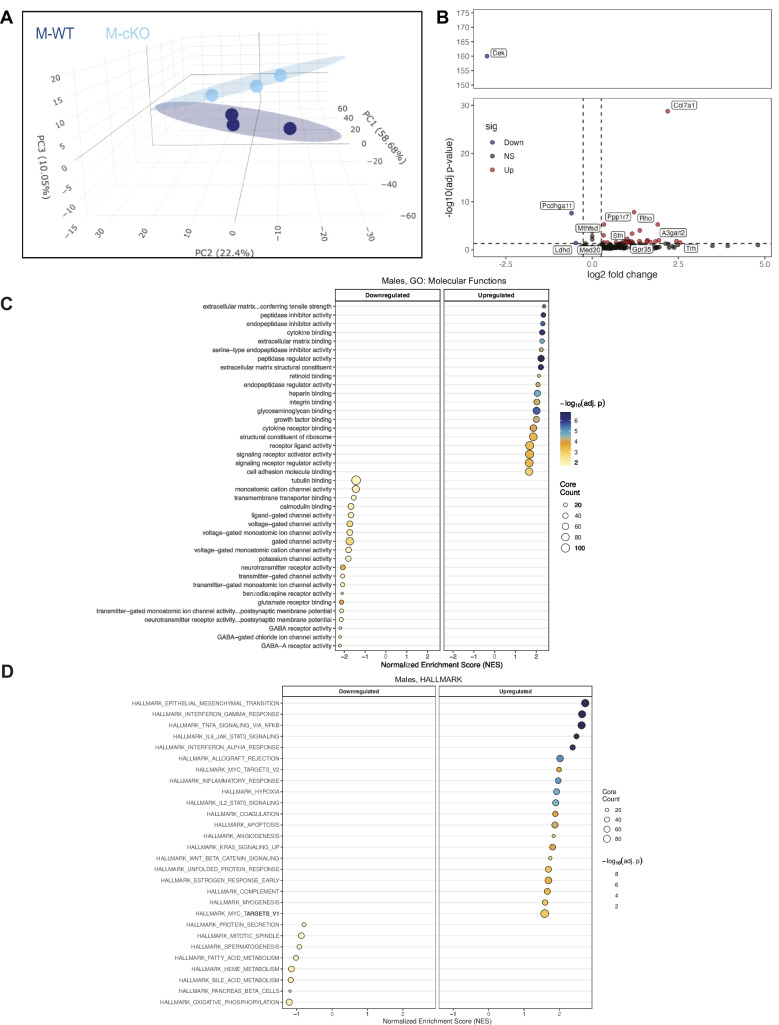
Differentially expressed genes in male *Dek* cKO mice are associated with upregulation of cytokine and inflammatory signaling, early estrogen response, and pro-survival pathways and downregulation of metabolism and neurotransmitter receptor activity. **A** Principal component analysis (PCA) shows the separation of male WT and *Dek* cKO mice due to variation in the transcriptome using hippocampal mRNA. The ellipses represent 95% confidence intervals. *N* = 3/genotype. **B** The volcano plot depicts genes upregulated (red) and downregulated (blue) in male *Dek* cKO hippocampus compared with WT mice. **C**,** D** Gene Set Enrichment Analysis for Molecular Functions (**C**) and Hallmark signaling (**D**) using DeSeq2-identified differential gene expression. Dot size represents the number of genes in the gene set differentially expressed in *Dek* cKO tissue vs WT tissue. The color scale represents *p* value, with light yellow indicating lower *p* values. The *X* axis graphs the gene set enrichment based on Normalized Enrichment Score (NES). Negative NES values represent down-regulation of a gene set and positive NES values depict up-regulation of a gene set

We next evaluated the transcriptomes of males and females separately. *Dek* cKO and WT male transcriptomes were clearly separated by PCA, with PC1 accounting for 58.68% of the variance (Fig. 6A). There were 32 DEGs (adj *p* value < 0.05, ± 1.2-fold change), with 29 genes upregulated and only 3 genes downregulated, including *Dek* (Fig. 6B). When expanded to include raw *p *value < 0.05, ± 1.5-fold change, there were 456 upregulated DEGs and 69 downregulated DEGs. GSEA was used to evaluate the molecular functions impacted by *Dek* loss in the male hippocampus. Molecular functions upregulated upon *Dek* loss in males included extracellular matrix components, cytokine binding, peptidase inhibitor activity, and growth factor binding. Down-regulated molecular functions included neurotransmitter/glutamate/GABA receptor activity, tubulin binding, voltage gated channel activity, and monoatomic cation channel activity (Fig. 6C). GSEA identified up-regulation of Hallmarks of Myc gene targets, early estrogen response, Wnt/β-catenin signaling, apoptosis, the unfolded protein response (UPR), and inflammatory responses including interferon gamma (IFNγ), TNFα, and Jak/Stat signaling. Hallmarks identified to be downregulated in the male *Dek* cKO transcriptome included fatty acid metabolism and mitotic spindles (Fig. 6D).

**Fig. 7:**
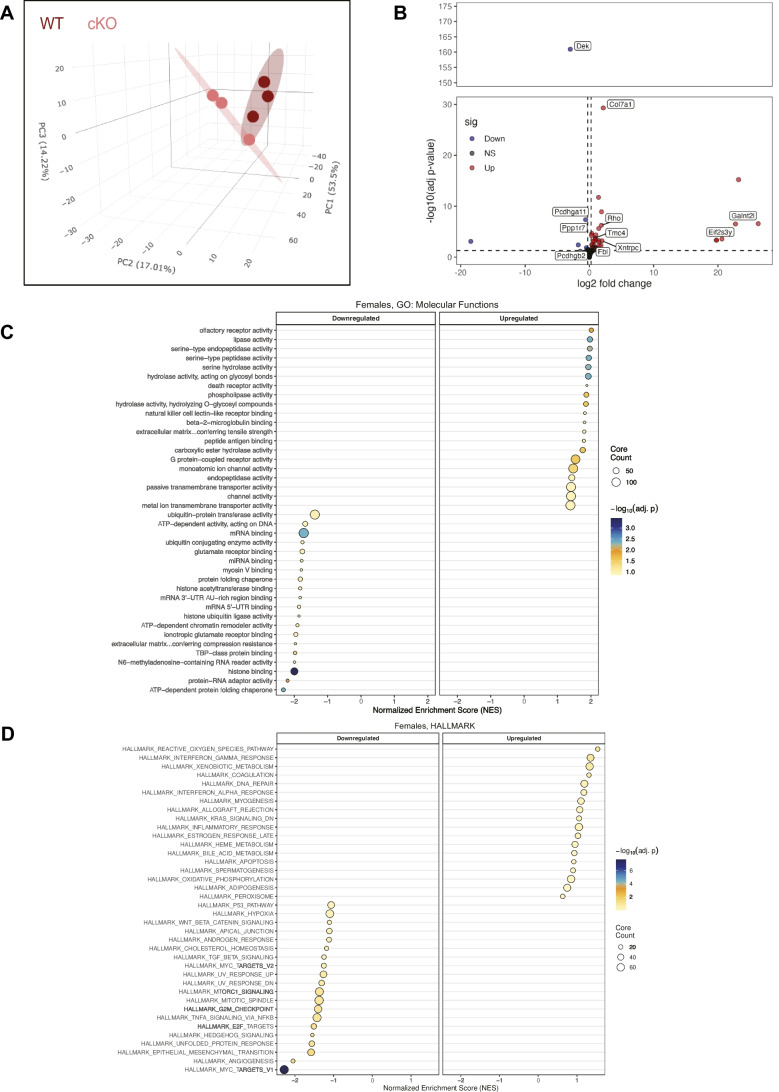
Differentially expressed genes in female *Dek* cKO mice are associated with upregulation of cytokine and inflammatory signaling, peptidases, and p53/DNA repair and downregulation of cell cycle and proteotoxic stress response. **A** Principal component analysis (PCA) shows the separation of female WT and *Dek* cKO mice due to variation in the transcriptome using hippocampal mRNA. The ellipses represent 95% confidence intervals. *N *= 3/genotype. **B** The volcano plot depicts genes upregulated (red) and downregulated (blue) in male *Dek* cKO hippocampus compared with WT mice. **C**, **D** Gene Set Enrichment Analysis for Molecular Functions (**C**) and Hallmark signaling (**D**) using DeSeq2-identified differential gene expression. Dot size represents the number of genes in the gene set differentially expressed in *Dek* cKO tissue vs WT tissue. The color scale represents *p *value, with light yellow indicating lower *p* values. The *X* axis graphs the gene set enrichment based on Normalized Enrichment Score (NES). Negative NES values represent down-regulation of a gene set and positive NES values depict up-regulation of a gene set

We next evaluated female *Dek* cKO transcriptomes versus WT females. Principal component analysis showed separation of female *Dek* cKO hippocampal transcriptomes from female WT, with separation primarily along the PC1 axis (53.5% of variance, Fig. 7A). In *Dek* cKO females, there were 46 DEGs (adj *p *value < 0.05 ± 1.2-fold change), with 40 upregulated and 6 downregulated, including *Dek* (Fig. 7B). When expanded to include raw *p* value < 0.05 ± 1.5-fold change DEGs, there were 307 upregulated genes and 100 downregulated genes. GSEA to identify impacted molecular functions revealed few similarities when compared with males. Like males, female *Dek* cKO hippocampal transcriptomes indicated downregulation of glutamate receptor binding and upregulation of extracellular matrix components. However, unique to females were the upregulated molecular function of death receptor activity and downregulated molecular functions of mRNA binding, protein folding chaperones and ubiquitin conjugation, and histone binding (Fig. 7C). GSEA for Hallmark gene sets revealed upregulation of estrogen late responses, DNA repair, the p53 pathway, and apoptosis. Downregulated Hallmark gene sets included androgen response, cholesterol homeostasis, and cell cycle-relevant categories such as mitotic spindle, E2F targets, and G2/M checkpoints (Fig. 7D). Male and female *Dek* cKO transcriptomes both had upregulation of the Hallmark gene sets for IFNγ and inflammatory responses and downregulation of mitotic spindle gene sets. This indicates a shared effect of *Dek* loss on potentially increasing neuroinflammation and decreasing glutamate receptor binding, while female *Dek* cKO mice had increased transcriptomic evidence of stress responses, including proteotoxic stress, DNA repair, and the p53 pathway.

**Fig. 8:**
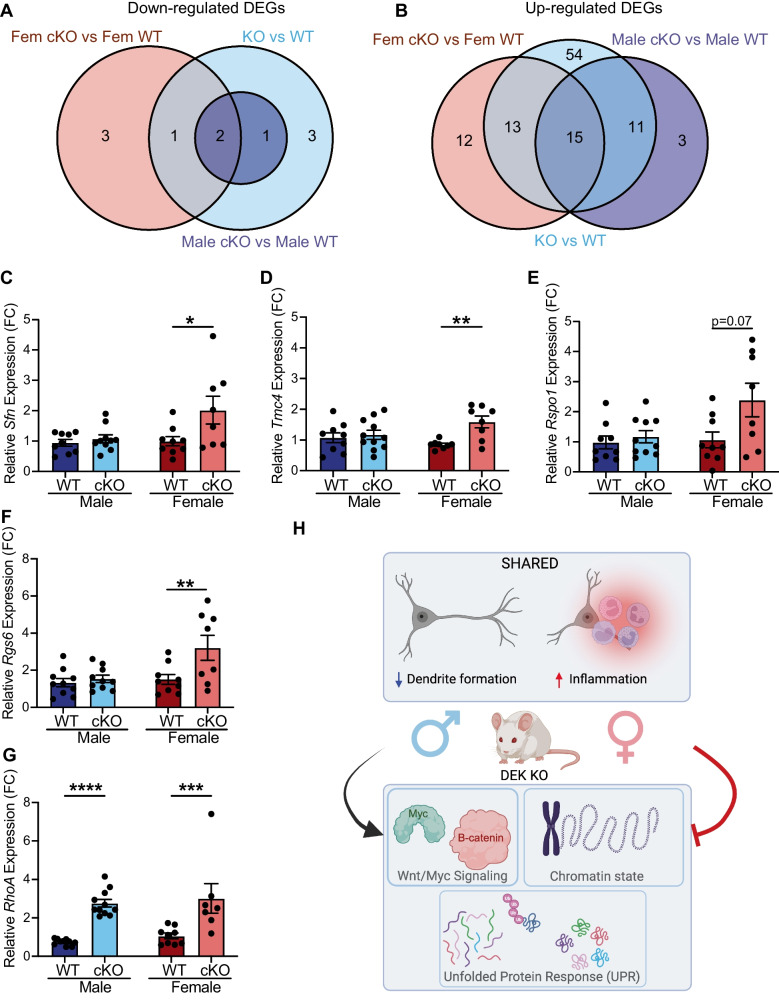
Male and female *Dek* cKO hippocampi have shared and unique differentially expressed genes. **A**–**B** Venn diagrams depict the number of downregulated (**A**) and upregulated (**B**) DEGs in female vs. male *Dek* cKO mice and compared to the (sex combined) all cKO mice. **C**–**G** Quantitative RT-PCR was used to validate select DEGs in male and female wild-type and *Dek* cKO hippocampal tissue. Female *Dek* cKO mice were the only group to upregulate *Sfn* (**C**), *Tmc4* (**D**), *Rspo1* (**E**), and *Rgs6* (**F**). Both male and female *Dek* cKO showed increased expression of *RhoA* (**G**)*.* The mean ΔCT value for all wild-type controls (male + female) was used for normalization to calculate ΔΔCT values for each sample, to depict expression variability of target genes among control samples. Unpaired *t*-tests were used to compare within-sex gene expression differences between WT and *Dek* cKO samples. * *p* < 0.05, ** *p* < 0.01, ***p < 0.001, **** *p* < 0.0001. **H** Graphical abstract summarizing transcriptomic data of DEGs for male and female *Dek* cKO mice. Both male and female *Dek* cKO have transcriptomic signatures of inflammation, immune responses, and downregulated gene sets related to dendrite morphogenesis. However, male *Dek* cKO mice have a transcriptome that indicates upregulated Wnt/β-catenin and Myc signaling, as well as the unfolded protein response. In contrast, female *Dek* cKO mice show evidence of inhibited Wnt/β-catenin and Myc signaling, the unfolded protein response, and histone/chromatin binding. Image made with BioRender

Most interestingly, there were several Molecular Function and Hallmark gene sets that were regulated in opposite directions between male and female *Dek* cKO mice. This likely contributed to their separation on the PCA plot (Fig. 5A). Notably, males upregulated peptidase inhibitors while females increased serine peptidases, as well as hydrolases and lipases (compare Figs. 6C and 7C). This correlates with differences in Hallmark gene sets, where males upregulated the unfolded protein response while females downregulated the unfolded protein response (compare Figs. 6D and 7D). This downregulated UPR, along with downregulated protein folding chaperones, may lead to the need to increase peptidases for degradation of misfolded proteins. In addition, where males showed upregulated gene sets related to Wnt/β-catenin signaling, mTor, and Myc targets, these were downregulated in females. In contrast, males downregulated oxidative phosphorylation while this was an upregulated Hallmark in females. A comparison of the DEGs for each sex revealed that male and female *Dek* cKO hippocampal transcriptomes shared 2 downregulated genes (*Dek* and *Pcdhga11*) and 15 upregulated genes (e.g., *Col7a1*, *RhoA, Ovgp1, Galnt2l, Sbk3, Mthfsd, Npcd, Dpm1, Palmd,* and *Tnfrsf23*) (Fig. 8A, B). Interestingly, there were > 50 genes that reached significance when male and female *Dek* cKO samples were evaluated together, suggesting that the increased statistical power of 6 samples (vs 3/sex) yields additional shared DEGs between the sexes (Fig. 8A, B). The GSEA Biological Processes for both male and female *Dek* cKO samples were similar compared with the overall cKO analysis (compare Supplementary Fig. S5 with Fig. 5C).

Finally, we used quantitative RT-PCR to validate DEGs that were either shared between sexes or showed sex differences. We specifically chose genes relevant to the most significant molecular functions and hallmarks across a range of signaling mechanisms, including DNA damage and cell cycle (*Sfn*)*,* ion channels (*Tmc4*), sex differences and Wnt/β-catenin signaling (*Rspo1*), hippocampal neurogenesis and memory (*Rgs6*)*,* and dendrite morphogenesis (*RhoA*). In females, *Sfn* expression was significantly increased in *Dek* cKO mice compared with WT controls (*t*(8.65) = 2.21, *p* < 0.05; Fig. 8C), whereas no genotype effect was observed in males. Female *Dek* cKO mice also showed elevated expression of *Tmc4* (*t*(14.87) = 2.08, *p* < 0.05; Fig. 8D), a strong trend toward increased *Rspo1* expression (*t*(9.31) = 1.97,* p* = 0.07; Fig. 8E), and significantly increased *Rgs6* expression (*t(*9.09) = 2.34, *p* < 0.05; Fig. 8F), with no corresponding genotype-dependent differences detected in males for any of these genes. In contrast to these female *Dek* cKO-restricted effects, *RhoA* expression was significantly increased in *Dek* cKO mice of both sexes (males: *t*(11.30) = 9.75, *p* < 0.05; females: *t*(6.15) = 2.30, *p* < 0.05; Fig. 8G) indicating a shared transcriptional response to *Dek* loss. Collectively, these findings demonstrate genotype-dependent transcriptional changes in *Dek* cKO mice, with several genes showing altered expression selectively in females although there is evidence of shared transcriptomic changes in both sexes. A summary of the transcriptomic findings is presented in Fig. 8H.

## Discussion

In the present study, a comprehensive behavioral battery and hippocampal transcriptomic profiling set of analyses was conducted to define the behavioral and molecular consequences of *Dek* loss in mice. We demonstrate that loss of *Dek* results in sex- and task-specific cognitive deficits, with females exhibiting a selective vulnerability characterized by impaired cognitive flexibility rather than generalized learning or other behavioral impairments. Whereas our prior work linked reduced DEK expression with cellular and molecular phenotypes related to Alzheimer’s disease and related dementias (AD/ADRD), as well as age-related cognitive decline, using in vitro approaches [9, 37], the present study provides the first evidence of the functional consequences of perturbed *Dek* expression in vivo within a learning- and memory-relevant brain region. We identified both shared and sex-specific transcriptomic alterations in the hippocampus of male and female *Dek* cKO mice when compared with male and female *Dek* WT mice. These changes highlight the molecular consequences of *Dek* loss, including alterations in pathways involved in inflammatory signaling, neuronal structural regulation, and transcriptional control. Together, these transcriptomic signatures suggest a broader role for DEK in supporting brain function and cognitive processes in a sex-dependent manner. Although these transcriptomic changes may be independent of the observed behavioral phenotype, they align with our previous findings implicating DEK in cellular and molecular processes that support neuronal vitality and cognitive resilience.

The female-specific vulnerability incognitive flexibility following *Dek* loss complements our prior work in humans indicating that postmenopausal women with schizophrenia-associated dementia exhibit progressively reduced DEK expression as dementia severity increases, whereas no corresponding changes were observed in men [28]. This relationship is further supported by the localization of the human *DEK* gene within a schizophrenia risk locus at chromosome 6p23 [38]. Taken together, these findings position DEK as a critical regulator of sex-specific cognitive resilience, whose disruption preferentially impacts executive-like cognitive domains in females rather than global impairments in learning or memory.

To more precisely interpret the behavioral phenotype associated with *Dek* loss, mice were evaluated in a variety of behavioral tasks probing partially dissociable cognitive, affective, and sensorimotor systems. This approach was designed to distinguish cognitive domain-specific impairments from global disruptions in activity or movement, motivation, or emotionality. The open field assay was used to assess baseline locomotor activity and anxiety-related exploratory behavior [39]. Novel object recognition was used to probe object-based memory and familiarity processing [31, 40]. Spatial learning, reference memory, and cognitive flexibility were evaluated using the Morris water maze, including a reversal phase that placed increased demands on strategy updating and executive control [41]. Acoustic and tactile startle responses, together with prepulse inhibition, were included to assess sensorimotor reactivity and gating [39]. Finally, contextual and cued conditioning were used to examine associative learning under emotionally salient conditions [42]. Evaluating performance across these complementary tasks allowed us to define the extent and specificity of behavioral consequences associated with *Dek* loss and to determine whether effects were broad or instead emerged selectively in a task- and potentially circuit-dependent manner.

Exploration and anxiety-related behavior assessed in the open field were intact, indicating that altered anxiety-like behaviors or gross locomotor impairment do not account for performance differences observed in more demanding tasks. Similarly, there were no differences in novel object recognition; however, this does not preclude potential differences when using alternative task parameters in this assay. During Morris water maze acquisition, both male and female *Dek* cKO mice acquired the spatial task at comparable rates compared with WT controls, with normal reductions in distance traveled, preserved path efficiency, and intact probe-based spatial accuracy, indicating largely preserved hippocampal-dependent spatial learning and reference memory. Although female *Dek* cKO mice exhibited modest increases in escape latency on later acquisition days and fewer platform site entries, distance and swim speed were comparable to WT on the probe trial, suggesting subtle to no alterations in memory. Sensorimotor processing was likewise intact, as acoustic startle responses did not differ by genotype, although female *Dek* cKO mice exhibited enhanced tactile startle reactivity accompanied by preserved or enhanced prepulse inhibition, indicating increased sensory responsiveness to tactile input that remains under effective inhibitory control rather than reflecting generalized disinhibition or dysregulated sensorimotor reactivity. Finally, contextual and cued conditioning revealed no genotype- or sex-dependent impairments, indicating preserved aversively motivated learning and memory. The relative preservation of performance across several performance domains highlights the selective and task-specific nature of the behavioral alterations observed in female *Dek* cKO mice.

Despite intact acquisition learning and memory across tasks, female *Dek* cKO mice exhibited a distinct impairment during the Morris water maze reversal learning phase. Reversal learning is a form of memory updating that requires animals to modify previously learned spatial associations when task contingencies change. This phase places demands on cognitive flexibility and on the ability to update and refine learned strategies rather than simply recall prior information [41].

During reversal learning, female *Dek* cKO mice showed increased escape latency and reduced path efficiency during late-phase acquisition. They also made fewer platform site entries during the reversal probe trial, despite preserved proximity to the platform location. This pattern indicates that spatial memory representations are intact, but that strategy change and goal-directed search were inefficient. Swim speed differences emerged late during reversal training (day 5), making it unlikely that motor factors account for the observed learning inefficiencies. Together, this behavioral profile may be characterized as disrupted cognitive flexibility rather than impaired spatial learning, motivation, or motor capacity.

Reversal learning engages executive control processes mediated by prefrontal regions, including the medial prefrontal and orbitofrontal cortices. These processes operate in coordination with hippocampal spatial representations and are commonly assessed as a measure of cognitive flexibility and adaptive updating of learned rules [43–45]. Although reversal learning places substantial demands on prefrontal executive systems, successful spatial reversal also requires hippocampal mechanisms that support the updating, refinement, and stabilization of newly acquired spatial representations. Consistent with this framework, the behavioral findings in the reversal learning phase of the Morris water maze suggest disruption of hippocampal–prefrontal circuitry. Based on known sex differences in DEK protein expression within hippocampal subfields [9] and the task’s reliance on flexible spatial memory updating, exploratory transcriptomic analyses were initially focused on the hippocampus to identify shared and unique candidate molecular and cellular pathways between males and females following *Dek* loss.

Overall, when compared with WT controls, only ~ 10% of differentially expressed genes in the hippocampus of *Dek* cKO mice were downregulated. The vast majority of DEGs were upregulated upon *Dek* loss. DEK promotes the formation of (closed) heterochromatin via multiple mechanisms to silence (down-regulate) gene expression. One mechanism involves facilitating the post-translational modification of histone H3 to create heterochromatin epigenetic marks, like H3K27me3, that compact chromatin to silence gene expression [30, 33, 46]. A second mechanism is through its ability to bind all three essential components of the nucleosome: the histone core, dyad DNA, and linker DNA. Through its nucleosome binding, DEK physically bends linker DNA, which likely leads to nucleosomes being pulled closer together [33, 46]. Therefore, it is expected that chromatin would become more open, and more accessible to transcription machinery, at specific genetic loci upon DEK loss through both physical nucleosome manipulation and histone H3 post-translational modification. This increased chromatin accessibility would lead to increased expression of target genes upon DEK loss, as we observed.

Generally, our transcriptomic data suggests that elimination of *Dek* negatively impacts neuronal function, neuron vitality, and tissue homeostasis. Regardless of sex, the transcriptomic data from *Dek* cKO mice had an exceptionally strong upregulation of gene sets for inflammation, immune responses to pathogens, and positive regulation of innate and adaptive immune responses. Relevant genes in this category included *IL21, IL11*, *Ccl7*, *Ccl2,* and *Ly6G-5b*. This is intriguing, because it is typically the over-expression, or aberrant extra-cellular localization, of DEK that is associated with immune system activation and inflammation. For example, extra-cellular DEK is detected in neutrophil extracellular traps (NETs), as a secreted chemotactic factor for immune cells, and as an autoantigen for autoimmune diseases like juvenile idiopathic arthritis [47–49]. Gene set enrichment for adaptive and humoral immune responses suggests that circulating lymphocytes may be able to more readily access the brain in *Dek* cKO mice. However, the constitutive loss of DEK protein may also contribute to the activation or dysfunction of microglia, which is feasible given the well-documented role of DEK in myelopoiesis [50–54]. Recently, it was reported that DEK interacts with IRE1α, a central regulator of endoplasmic reticulum (ER) stress [55], and that the loss of DEK exacerbates toxin-induced inflammation in the intestines by increasing ER stress. ER stress leads to an accumulation of misfolded proteins and triggers the unfolded protein response (UPR). Misfolded proteins and the UPR can both lead to, and be a consequence of, chronic inflammation. Notably, protein folding, chaperone proteins, and the UPR were both detected as enriched gene sets in *Dek* cKO mice but differed by sex. GSEA suggested that cells in the hippocampus of male *Dek* cKO mice upregulate the UPR, while females downregulated the UPR and showed downregulation of gene sets linked to ubiquitination and protein folding chaperones. Perhaps, as a compensatory mechanism, females may be upregulating peptidases to try to clear accumulating misfolded proteins. Evidence supporting that DEK loss may lead to protein accumulation, and aggregation includes multiple independent publications reporting that DEK-deficient human and mouse neurons demonstrate increased amounts of hyper-phosphorylated Tau and evidence of intracellular Tau aggregates [56, 57] that, in mice, correlate with microglia activation and neuron loss. It will be important for future work to validate that the loss of DEK protein results in neuroinflammation, and to determine whether the cause is via intrinsic effects on microglia, an accumulation of misfolded proteins or protein aggregates (e.g., Tau tangles, β-amyloid plaques, or other proteins) leading to inflammatory necroptosis, neuron/synaptic dysfunction, or through an unidentified mechanism. It will also be necessary to validate potential sex differences in proteotoxic stress, as indicated by our RNA-seq data.

Transcriptomic analyses also revealed the downregulation of gene sets associated with dendritic spines, dendrite morphogenesis, and synaptic structure and plasticity. We have previously reported that DEK-deficient human SH-Sy5y cells differentiated to neurons have shorter neurites, lending support to our transcriptomic data that *Dek* loss may lead to impaired dendrite formation and function. Importantly, we validated that *RhoA* is upregulated in *Dek* cKO hippocampal tissue in both males and females. RhoA not only functions in actin cytoskeleton organization, but also strongly restricts dendritic growth and branching, including in hippocampal neurons [58]. Furthermore, prior work demonstrated that *Dek*-silenced neurons show increased excitability, using patch-clamp analyses, and dysregulated expression of sodium ion channel genes [57]. Our transcriptomic data supports a role for DEK in regulating the expression of ion channels, including our qRT-PCR data confirming increased expression of *Tmc4*, a voltage-gated chloride channel, in female *Dek* cKO mice, as well as increased expression of ion channel genes *Clcn1*, *Kcna10*, and *Scnn1g* and down-regulation of the potassium ion channel gene *Kcng3* in the RNA-seq data. We additionally observed enrichment of gene sets pertaining to neurotransmitter, GABA and glutamate receptors. The transcriptomics data in this work strongly supports prior publications, which reported *Dek* loss led to detrimental changes in neuron morphology, including poor dendrite/neurite formation, and synaptic dysfunction, which may contribute to poor neuron vitality and/or excitotoxicity [24, 26].

We identified *Rgs6, Rspo1,* and *Sfn* (Stratifin or 14-3-3σ), to be significantly upregulated genes in female *Dek* cKO mice. The 14-3-3 family of scaffolding proteins interacts with phosphorylated proteins to control multiple signaling pathways, including mTor and NFκB signaling, the DNA damage-induced G2/M cell cycle checkpoint, apoptosis, and p53 stabilization in response to stress [59]. While knowledge regarding the role of 14-3-3σ in the brain is limited, it has been reported to be highly expressed in reactive astrocytes and to be upregulated in response to cellular stress. Elevated levels of 14-3-3 proteins have also been detected in brain tissue from patients with neurological conditions such as multiple sclerosis and ischemic stroke [60]. It has also been detected in Lewy bodies and shown to physically interact with Tau [61–63]. Its role in *Dek* cKO mice will be an interesting avenue of future research, and its upregulation is consistent with altered cellular stress response pathways in the DEK-deficient hippocampus.

Interestingly, *Rspo1* and *Rgs6* both have connections to sex differences. Rspo1 (R-spondin1) is a secreted protein required for ovarian differentiation and female sex development in utero [64] and modulates Wnt signaling [65]. In the brain, there are no reports of sex-specific activity of R-spondin1 yet. However, *Rspo1* was recently shown to promote proliferation of neural stem cells (NSCs) within the dentate gyrus region of the hippocampus and is expressed by astrocytes to activate Wnt signaling in neighboring NSCs [66]. Regulator of G-protein signaling 6 (RGS6) inhibits G protein receptor signaling through its function as a GTPase activating protein (GAP), resulting in the inactive GDP-bound state of G proteins [67]. RGS6 targets serotonin1A, μ opioid, and muscarinic acetylcholine 2 receptors; however, RGS6 also has GTPase-independent functions [68]. In breast cancer cells, RGS6 over-expression also leads to cell cycle arrest and apoptosis [69]. RGS6 has been implicated in hippocampal-dependent learning and memory through its regulation of adult hippocampal neurogenesis, neuronal maturation, and experience-dependent cognitive enhancement. Loss of RGS6 disrupts exercise-induced improvements in hippocampal learning and memory, and reduced RGS6 expression has been associated with cognitive impairment in Alzheimer’s disease models [70, 71]. Interestingly, female, but not male, mice with loss of *Rgs6* in dopamine neurons exhibited decreased binge-like alcohol consumption [72]. In contrast, the *increased Rgs6* expression observed in the hippocampus of female *Dek* cKO mice suggests that DEK loss may alter RGS6-associated signaling in a context- and sex-dependent manner. While these findings support a role for RGS6 in neural processes relevant to learning and memory, its contribution to executive functions and cognitive flexibility has not been clearly defined. Further, at this juncture, the role of any of the targeted genes in regulating the DEK-mediated effects on behavior in females is conjecture. However, collectively, transcriptomic analyses revealed several molecular pathways that may contribute to the observed behavioral differences, including inflammation, dendrite morphogenesis, proteotoxic stress, and synaptic function.

A primary limitation of the present study is the use of the CMV-Cre promoter, which results in whole-brain deletion of *Dek* irrespective of cell type or region. Future studies employing tissue- and cell-specific deletion strategies will be necessary to determine whether specific cell populations drive discrete components of the *Dek* cKO transcriptomic and behavioral phenotypes. In addition, the mean age of the mice (6–8 months) suggests that some females may not yet have reached estropause, which typically occurs between 9 and 12 months of age in rodents [73]. Thus, future work should examine older cohorts to better account for the combined effects of aging and declining ovarian hormone levels. Nevertheless, we do not suspect that estrous cycle stage substantially influenced *Dek* expression in younger females, despite *Dek* being an ERα target gene, as *Dek* mRNA levels were remarkably consistent across females that were neither age- nor estrous-matched. Given that DEK is highly expressed during fetal brain development [27], we also cannot exclude the possibility of developmental contributions to the observed phenotypes in this constitutive knockout model.

Additional limitations include limited statistical power to directly compare males and females using two-way ANOVA and the correlative nature of the transcriptomic data, which precludes definitive causal interpretation. Confirmation that protein levels are, indeed, different for the identified DEGs and additional mechanistic studies both will be required to determine which molecular pathways downstream of *Dek* loss directly contribute to the sex-specific cognitive differences reported here. An analysis of the chromatin state in *Dek*-deficient brains will also be informative. Another limitation is the focus on the hippocampus as the sole region for transcriptomic analyses. Again, given the behavioral phenotype observed during the reversal phase of the Morris water maze, brain regions beyond the hippocampus are also likely to be involved, including the medial prefrontal cortex (mPFC) [74].

To our knowledge, this is the first study to directly assess the consequences of *Dek* deficiency in the brain on learning and memory using in vivo behavioral analyses. Although prior work has linked DEK loss to molecular and cellular features associated with neurodegeneration and cellular/molecular hallmarks of AD/ADRD [24, 26, 28], its functional impact on cognition had not been examined. Here, we demonstrate sex-specific effects of *Dek* loss on cognitive performance, with vulnerability in tasks that place demands on executive function. While hippocampal transcriptomic changes may not directly account for the observed behavioral phenotype, they provide important insight into the cellular and molecular pathways affected by DEK loss and advance our understanding of its role in brain function. Future studies incorporating prefrontal regions, circuit-level analyses, and additional executive control tasks will be necessary to determine how these molecular alterations relate to sex-dependent cognitive flexibility. Together, these findings position *Dek* cKO mice as a useful model for investigating sex- and cognitive domain-specific mechanisms of vulnerability and resilience, with particular relevance to executive function and cognitive flexibility.

## Materials and Methods

### Mouse Model

Handling of mice was performed with the approval of the Cincinnati Children’s Institutional Animal Care and Use Committee and approved under protocols 2020–0037, 2023–0043, and 2023–0022. Mice were housed in a controlled environment with a 12-h light/12-h dark cycle in specific pathogen-free housing, with free access to water and a standard irradiated chow diet (Lab Diet, Richmond, VA, USA) and RO and UV sterilized water.

The *Dek* conditional knockout allele was generated using the CRISPR technology to introduce 5’ and 3’ loxP sites sequentially to flank exons 3 and 4 of this gene as described [30]. *Dek *floxed mice were donated to The Jackson Laboratory and are available as stock #041852. To test for phenotypes caused by *Dek* loss, *Dek*^*fl/fl*^ mice were bred to CMV-Cre mice on the C57Bl/6 background (Jackson Labs stock #006054). Once the deletion allele achieved germline transmission, the CMV-Cre transgene was eliminated from the colony by directed breeding, and the strain was maintained as a constitutive KO line via mating of heterozygous (*Dek*^+/Δ^) males and females. For behavioral studies, mice were 4–12 months old, while transcriptomic analyses were from mice 6–12 months old.

### Genotyping

Tail clips were digested with DirectPCR Lysis Reagent (Viagen Biotech) containing 0.6 mg/mL Proteinase K (Invitrogen). Samples were lysed for 6 h at 55 °C then 45 min at 85 °C. For PCR analysis, 1 μL of DNA was added to DreamTaq (Thermo Fisher) using the manufacturer’s specifications, with 0.2 μl of a 10-mM stock for each primer. All primers were synthesized by Integrated DNA Technologies (“IDT”). All PCR melting/denaturation temperatures were 94 °C and elongation temperatures of 72 °C for 1 min each cycle. Genotyping used a touchdown method, with *Cre* annealing temperatures of 60 °C (× 3 cycles), 58 °C (× 3 cycles), and 56 °C (× 26 cycles). *Dek* genotyping used annealing temperatures of 54 °C (× 3 cycles) and 52 °C (× 32 cycles). Samples were analyzed on a 3% agarose gel at 100 V for ~ 45 min.

*Dek* conditional knockout models were genotyped with the following primers:

- CMV-Cre:
  - Forward: GCGGTCTGGCAGTAAAAACTATC
  - Reverse: GTGAAACAGCATTGCTGTCACTT

*Dek* (detects endogenous and floxed alleles):

- 5′loxP Forward: AGTGAAATTACTGGTCTGTGAAG
- 5′loxP Reverse: CTGAGTGGAACAGCTCCTATAG
- 3′loxP Forward: AGATGCTTCACCTTAGAGCTG
- 3′loxP Reverse: TCAGTTTGGAGCAAATTTCATTTCC

*Dek* deletion allele:

- Forward (5′loxP forward): AGTGAAATTACTGGTCTGTGAAG
- Reverse (3′loxP reverse): TCAGTTTGGAGCAAATTTCATTTCC

### Behavioral Testing

A total of 58 mice (14 male *DEK* cKO, 22 male WT, 10 female *DEK* cKO, and 12 female WT) were tested in the Animal Behavioral Facility (RRID:SCR_022621) by personnel blinded to genotype.

#### Locomotor Activity/Open Field Test

To complete the open field test [75], mice were placed in SDI activity chambers (41 cm × 41 cm) for 60 min. Activity was monitored by the PAS system using PASUSB (v1.0.1.0) software (San Diego Instruments, San Diego, CA, USA) which records total beam breaks, ambulation (successive beam breaks) as well as center vs peripheral activity. Chambers were cleaned with Process NPD disinfectant (Steris Corp., St. Louis, MO, USA), an EPA approved, non-toxic denaturing, anti-bacterial, and anti-viral agent, between the evaluation of every mouse.

#### Morris Water Maze

The apparatus for the Morris water maze (MWM) [39, 75] consisted of a 150 cm diameter, 51 cm high, white tank with a 10 cm platform submerged 1–1.5 cm below water level. Water temperature was 21 ± 1 °C. Data were collected using video tracking software (AnyMaze v7.49, Stoelting Company, Wood Dale, IL, USA). The procedure had four phases. For all phases mice were tested in rotation with ~ 10 min intertrial intervals [42]. Mice that failed to find the platform were removed and placed on the platform for 10 s.

Phase 1 was training with curtains surrounding the tank. A 10-cm platform with an orange ball (10 cm above the water) was attached via brass rod to the platform. Each trial was limited to 90 s or when the mouse found the platform. The start and platform locations were identical for each trial. Mice that failed to find the platform twice within the six trials were retested 4–6 h later. Mice that failed during retesting were not included in subsequent phases.

Phase 2 was acquisition for 6 days and began the day after training. Curtains were opened to reveal distal cues positioned on the walls. A 10 cm submerged white platform was used. There are 5 days of learning and 1 memory (probe) trial on day 6. The mice underwent four trials each day with a 90-s limit per trial. There were 4 start positions (2 cardinal and 2 ordinal positions around the perimeter) used each day in a quasi-random order with the platform located in the same place on each trial. On day 6, the platform was removed, and a single 45 s trial was given with the mouse starting from a novel location. Acquisition provides data on distal cue learning, whereas the probe trial tests reference memory.

Phase 3, reversal, started the day after the acquisition probe trial. For this phase, curtains were opened, and a 7 cm platform was used with the platform in the opposite quadrant from acquisition. Again, there were 5 days of learning and a probe trial on day 6. These trials provide a test of cognitive flexibility.

Phase 4 was with a visible cue, but this time the position of both the start and platform was changed on each trial (4 trials/day for 2 days). Curtains were closed around the tank to obscure distal cues.

#### Acoustic and Tactile Startle (ASR-TSR)

Acoustic startle (ASR) and tactile startle responses (TSR) were assessed using a SR-LAB apparatus (San Diego Instruments, San Diego, CA, USA) with SR_LAB v1.0.1.2 software. Mice were placed in acrylic cylindrical holders mounted to an acrylic base with insulated legs with a piezoelectric accelerometer attached to the underside to detect movement [39]. The platform was positioned inside a sound-attenuated cabinet, and the system was calibrated daily. The session began with 5 min of habituation with no stimulus. Startle was assessed for 3 days. Days 1 and 2 consisted of 100 trials of alternating blocks of 5 acoustic and 5 tactile trials with intertrial intervals of 20 s. The acoustic stimulus was a 20 ms, 120 dB (SPL) mixed frequency white noise sound burst with 1.5 ms rise time. The tactile stimulus was a 20 ms 60 psi air puff, delivered to the dorsal surface of the mouse through a tube inserted in the top of the animal holder. Prepulse inhibition (PPI) of startle was assayed on day 3. Each mouse received a 10 × 10 Latin square sequence of 5 trial types (200 trials), with prepulses of 0, 59-, 70-, 80-, or 93-dB sound bursts lasting 20 ms for both TSR and ASR with a 50 ms gap before onset of the pulse, either acoustic or tactile. The dependent measure was the maximum startle response (*V*_max_) measured in mV.

#### Conditioned Freezing

Conditioned freezing (aka: conditioned fear) was assessed with the Freeze Monitor (San Diego Instruments, San Diego, CA, USA) in 25 cm × 25 cm test boxes as previously described [42]. Data were collected with Freeze Monitor System software v2.2.1. Each acrylic chamber had speakers mounted on the underside of the lid and a grid floor connected to a foot shock generator. Habituation was on day 1, in which mice were placed in the apparatus for 10 min. On day 2, the conditioning phase, mice were placed in the chamber for an acclimation period of 6 min where there were no stimuli, followed by 6 conditioned stimulus (CS)-unconditioned stimulus (US) pairings consisting of a tone and light with foot shock. The CS was an 85 dB, 2 kHz tone concurrent with the house light turning on. Each pairing consisted of the 30 s CS accompanied by foot shock (1.3 mA, though the gird floor) during the last 2 s. CS-US pairings were separated by 30 s intervals. On day 3, mice were returned to the chamber for 6 min with no stimuli and no shock and activity and freezing episodes were recorded as an index of contextual memory. On day 4, cued memory was tested; mice were placed in a different chamber made of black acrylic, hexagonal in shape with a solid floor. Mice were given 3 min of no stimuli followed by 3 min with light-tone without shock. This was followed by 10 extinction trials consisting of alternating light-tone intervals and 30 s of no stimuli. Activity and freezing were analyzed.

### Behavioral Software

Open field testing was conducted using San Diego Instruments PAS USB software (version 1.0.1.0). Morris water maze experiments were performed and analyzed using the AnyMaze Video Tracking System (version 7.49; Stoelting Co.). Acoustic startle response (ASR) and tactile startle response (TSR) assays were conducted using the SR-LAB Startle Response System (version 1.0.1.2; San Diego Instruments). Conditioned freezing behavior was assessed using the San Diego Instruments Freeze Monitor System (version 2.2.1).

#### RNA-seq

##### Tissue Collection

Mice were euthanized by isoflurane inhalation followed by decapitation. Brains were microdissected to remove the hippocampus and stored in RNA-Later at − 20 °C. Tissue was homogenized in 1 mL of Trizol using a tissue disruptor (Qiagen). Tissues were incubated on ice for 5 min, 200 μL of chloroform added, and the solution vortexed for 15 s. Then, the solution was incubated on ice for 14 min and vortexed halfway through. Samples were centrifuged at 12,000 rpm for 5 min at 4 °C. The aqueous phase was transferred to a 1.5 mL tube, and the DNA phase was frozen at − 80 °C. To precipitate RNA, 500 mL of 100% isopropanol alcohol was added to the aqueous phase. After inverting gently five times, the samples were incubated at room temperature for 10 min then centrifuged at 1200 × *g *for 15 min at 4 °C. The RNA pellet was washed in 1 mL of ice-cold 75% ethanol and then centrifuged for 15 min at 4 °C. The supernatant was removed and the pellet air dried in a sterile environment for up to 30 min, then reconstituted in RNase-free water.

##### Library Preparation and Sequencing

The RNA quality of each sample was checked with an Agilent 5300 Fragment Analyzer. All RNA samples had an RQN value between 8.1 and 9.6. As determined by InvitrogenTM QubitTM high-sensitivity spectrofluorometric measurement, 150 to 300 ng of total RNA was poly-A selected and reverse transcribed using Illumina’s TruSeq® stranded mRNA library preparation kit. Each sample was fitted with one of 96 adapters containing a different eight base molecular barcode for high-level multiplexing. After 15 cycles of PCR amplification, completed libraries were sequenced on an Illumina NovaSeqTM 6000 with a read depth of 20 million 100 bp paired-end reads per sample.

##### RNA-seq Analysis

Bulk RNA sequencing data were processed using the nf-core/rnaseq pipeline (v3.20.0) executed with Nextflow (v25.04.4). Adapter trimming and quality filtering were performed using Trim Galore (v0.6.10) with Cutadapt (v4.9). Ribosomal RNA contamination was removed using SortMeRNA (v4.3.7), and taxonomic contaminant screening was performed with Kraken2 (v2.1.5). Reads were aligned to the mouse reference genome (GRCm38/mm10) using STAR (v2.7.11b), and transcript abundance was quantified using Salmon (v1.10.3). Gene-level counts were generated using featureCounts (Subread v2.0.6). Additional quality control metrics were generated using FastQC (v0.12.1), Qualimap (v2.3), RSeQC (v5.0.2), dupRadar (v1.32.0), and MultiQC.

Transcript-level quantifications were imported into R (v4.4.2) using tximport (v1.32.0) and summarized to gene-level counts. Differential gene expression analysis was performed using DESeq2 (v1.44.0), incorporating models accounting for genotype and sex, including interaction terms where appropriate. Log2 fold change shrinkage was performed using the apeglm method. Variance-stabilizing transformation (VST) was applied for downstream visualization and principal component analysis (PCA).

PCA was performed on VST-transformed expression values to assess sample clustering. Confidence ellipses representing 95% confidence intervals for each group were drawn for PCA analyses: FemKO (pink), FemWT (red), MaleKO (light blue), and MaleWT (blue). Two parallel analyses were then performed: a two-group analysis comparing all *Dek* knockout (cKO) samples with all wild-type (WT) samples and a four-group analysis comparing *Dek*cKO vs. WT split into male and female. Differentially expressed genes (DEGs) were identified using thresholds of adjusted *p* value < 0.05 and absolute log2 fold change > log2(1.2). Visualization of DEGs included volcano plots, heatmaps, DEG count bar plots, and Venn diagrams, generated using R packages including ggplot2 (v4.0.1), ComplexHeatmap (v2.20.0), pheatmap (v1.0.13), and VennDiagram (v1.8.2).

Gene set enrichment analysis (GSEA) was performed using clusterProfiler (v4.12.6) and ReactomePA (v1.48.0), with gene rankings derived from DESeq2 Wald statistics. Gene identifiers were mapped using org.Mm.eg.db (v3.19.1) and AnnotationDbi (v1.66.0), and gene sets were obtained from msigdbr (v25.1.1). Enrichment results were visualized using enrichplot (v1.24.4). The data that support the findings of this study are available on the NCBI Gene Expression Omnibus (GEO) under accession number GSE308250.

#### RT-qPCR

One microgram of total RNA was reverse transcribed using the QuantiTect Reverse Transcription Kit (Qiagen) to generate cDNA. Quantitative Real-Time PCR was performed using SYBR Green PCR Master Mix on an ABI 7500 Real-Time PCR System (Applied Biosystems). Gene expression levels were normalized to the housekeeping gene PPIA and analyzed using the ΔΔCT method. To demonstrate the variability between control (WT) animals, the ΔCT value is averaged across all WT mice for a given gene, and that average ΔCT value is used for normalization to calculate ΔΔCT. This results in the mean WT (control) normalized value being ~ 1 but the inter-animal variability is shown. Primer sequences were used at a final concentration of 0.4 ng/μL each and included the following primers:

- mDek F: 5′-AACGTGGGTCAGTTCAGTGGC-3′
- mDek R: 5′-TTCGCTGTTCACGCCTGACCT-3′
- mPPIA F: 5′-GCGTCTSCTTCGAGCTGTT-3′
- mPPIA R: 5′-RAAGTCACCACCCTGGCA-3′
- mRgs6 F: 5’-AACACGGACTATGCCATCTATC-3′
- mRgs6 R: 5’-CCTCTGGAGTCTTGCTAAGTTT-3′
- mRspo1 F: 5’-CTGTGGCCCAGCTGTATATT-3′
- mRspo1 R 5’-CAGAAGGTTCCACTCCTTAGTT-3’.
- mRhoA F 5′-CAGAAGGCAGAGAAGGAAGTC-3′
- mRhoA R 5′-CTGGTGGGTGAAGATGTAGAAG-3′
- mSfn F 5′-ATGGACATCAGCAAGAAGGAG-3′
- mSfn R 5′-GTTGGCTATCTCGTAGTGGAAG-3′
- mTmc4 F 5′-GATCGTCTTGATCCTGCTTAGG-3′.
- mTmc4 R 5′-CATCCTTCTGCCTCCATGTT-3′

#### Statistical Procedures

Due to the sample size, behavioral data were analyzed separately for each sex using generalized linear mixed-effects models (SAS Proc Mixed, SAS Institute 9.4 TS, Cary, NC, USA) [76] or *t*-tests. Analyses included genotype as the main effect, with repeated-measure factors defined as 5-min time bins (open field), blocks of five trials (ASR/TSR), trial type (ASR/TSR with acoustic prepulses), individual trials (straight water channel, MWM cued training, and conditioned freezing), or training day (MWM), as appropriate. Repeated-measure factors were fit to autoregressive moving average or autoregressive [77] depending upon best fit of the corrected Akaike Information Criterion. Significant interactions were analyzed using the slice option in Proc Mixed since it controls the overall error term in the analyses. The estimation method for the covariance parameters was by the restricted maximum likelihood method. The data were analyzed by mixed linear ANOVA (SAS Proc Mixed, SAS Institute 9.4 TS, Cary, NC, USA) and used Kenward-Rogers adjusted degrees of freedom, which can be fractional. A folded F test was conducted prior to *t*-tests to determine if variances were equal and if not, the Satterthwaite method was used. Grubb’s test was used to detect any outliers. Statistical significance was set at *p* ≤ 0.05 and error bars depict SEM.

## Supplementary Information

Below is the link to the electronic supplementary material.

Supplementary Material File 1 (DOCX 39.8 MB)

Supplementary Material File 2 (XLSX 7.56 MB)

## Data Availability

The data that support the findings of this study are available from the corresponding author (LMPV) and are available on the NCBI Gene Expression Omnibus (GEO) under accession number GSE308250.
