# Supplementary material for: DEK Loss Induces Task-specific Deficits in Learning and Memory and Reprograms the Hippocampal Transcriptome in Mice
Source: Mol Neurobiol. 2026 Jun 29;63(1):734. doi: 10.1007/s12035-026-06022-4 (PMC13314828; doi:10.1007/s12035-026-06022-4)
Supplement: Supplementary file 1 — Supplementary Material File 1 (DOCX 39.8 MB) [file 12035_2026_6022_MOESM1_ESM.docx]

**Supplemental Figures**

**
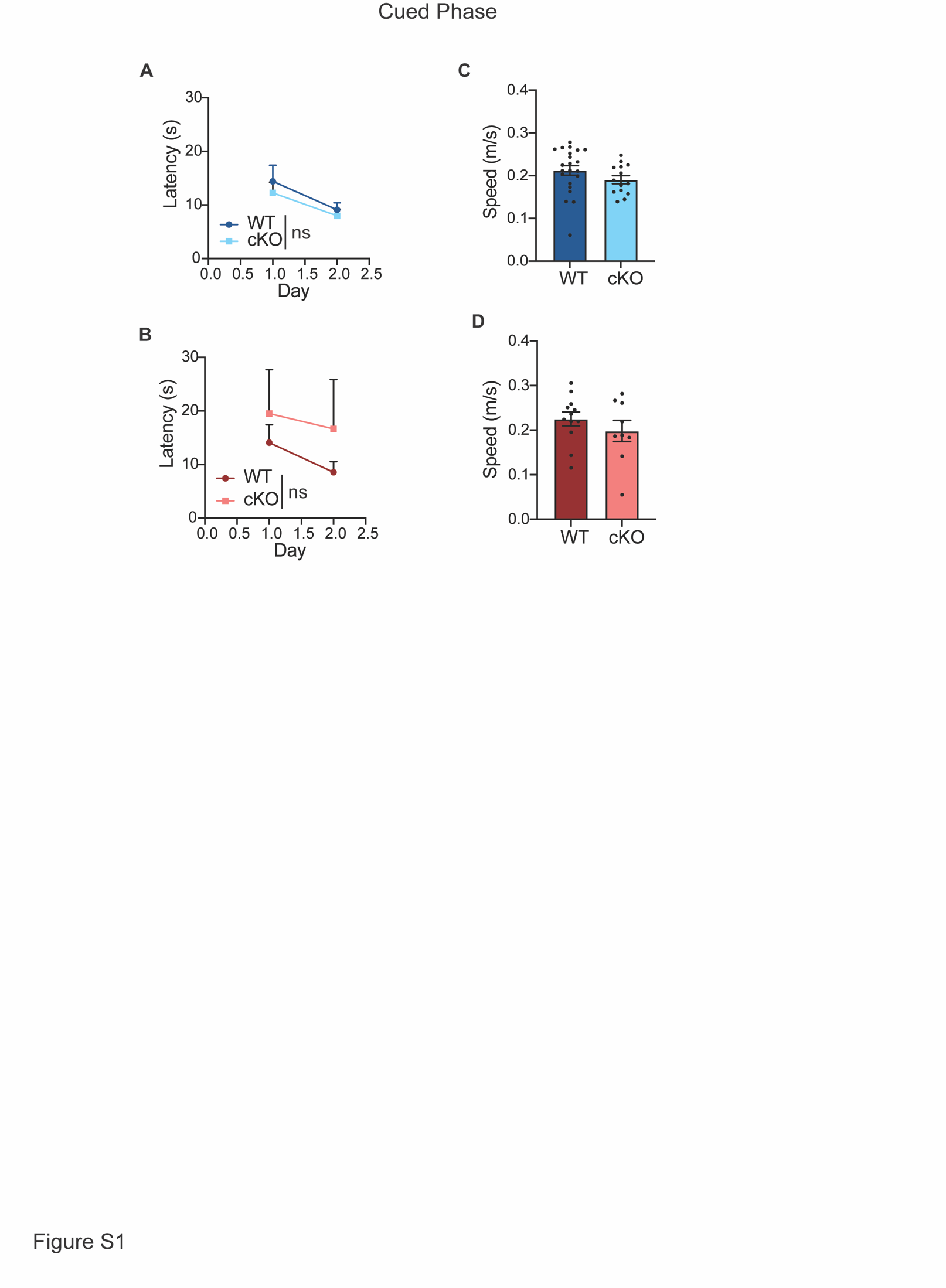
**

**Figure S1. DEK loss does not impact cued performance during reversal learning in the Morris Water Maze.** Cued Morris water maze performance was not impacted by DEK loss, with no genotype differences in escape latency (**A, B**) or swim speed (**C, D**) in either males (A, C; blue) or females (B, D; pink/red). Adult mice were used for the behavioral analyses: N=12 WT females, 10 *Dek* cKO females, 22 WT males, 14 *Dek* cKO males. Data are presented as mean ± SEM with individual data points shown.

**
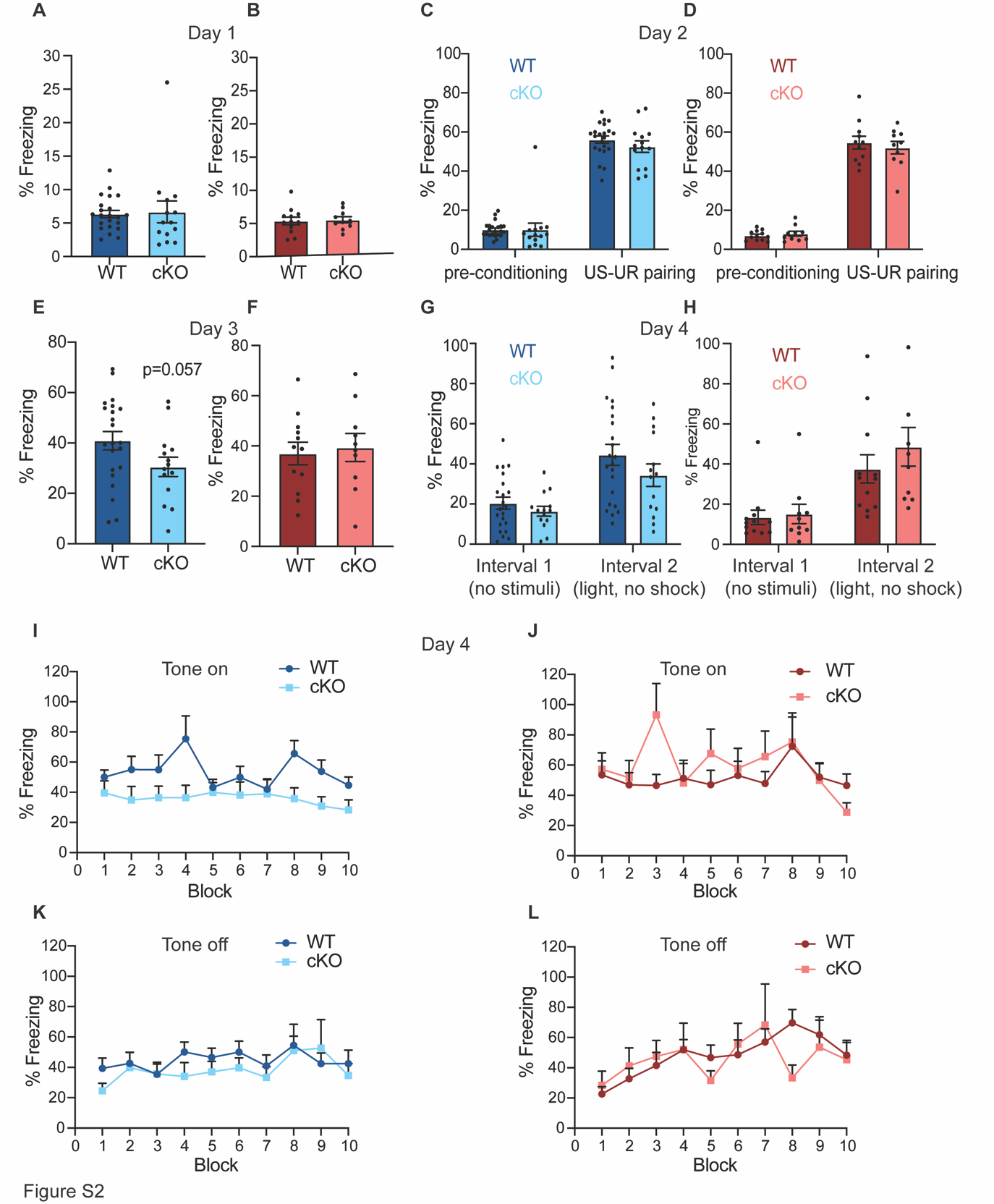
**

**Figure S2:** **Dek cKO mice have no consistent difference in conditioned fear learning memory.** Fear conditioning was assessed across acquisition and testing days in male (blue) and female (pink/red) mice. (**A–B)** Baseline freezing during Day 1 acquisition was comparable between genotypes in males (**A**) and females (**B**). (**C–D**) Freezing during US–UR pairings on Day 2 did not differ between WT and cKO males (**C**) or females (**D**). (**E–F**) On Day 3, male cKO mice exhibited reduced freezing compared to WT, representing a strong trend toward significance (p = 0.057; E), while no genotype differences were detected in females (**F**).
(**G–H**) Freezing during cued testing on Day 4 across stimulus intervals was similar between genotypes in males (**G**) and females (**H)**. (**I–L**) Tone‑on (**I, J**) and tone‑off (**K, L**) freezing across blocks on Day 4 did not reveal significant genotype‑dependent differences in either sex. Adult mice were used for the behavioral analyses: N=12 WT females, 10 *Dek* cKO females, 22 WT males, 14 Dek *cKO* males. Data are shown as mean ± SEM with individual data points shown.


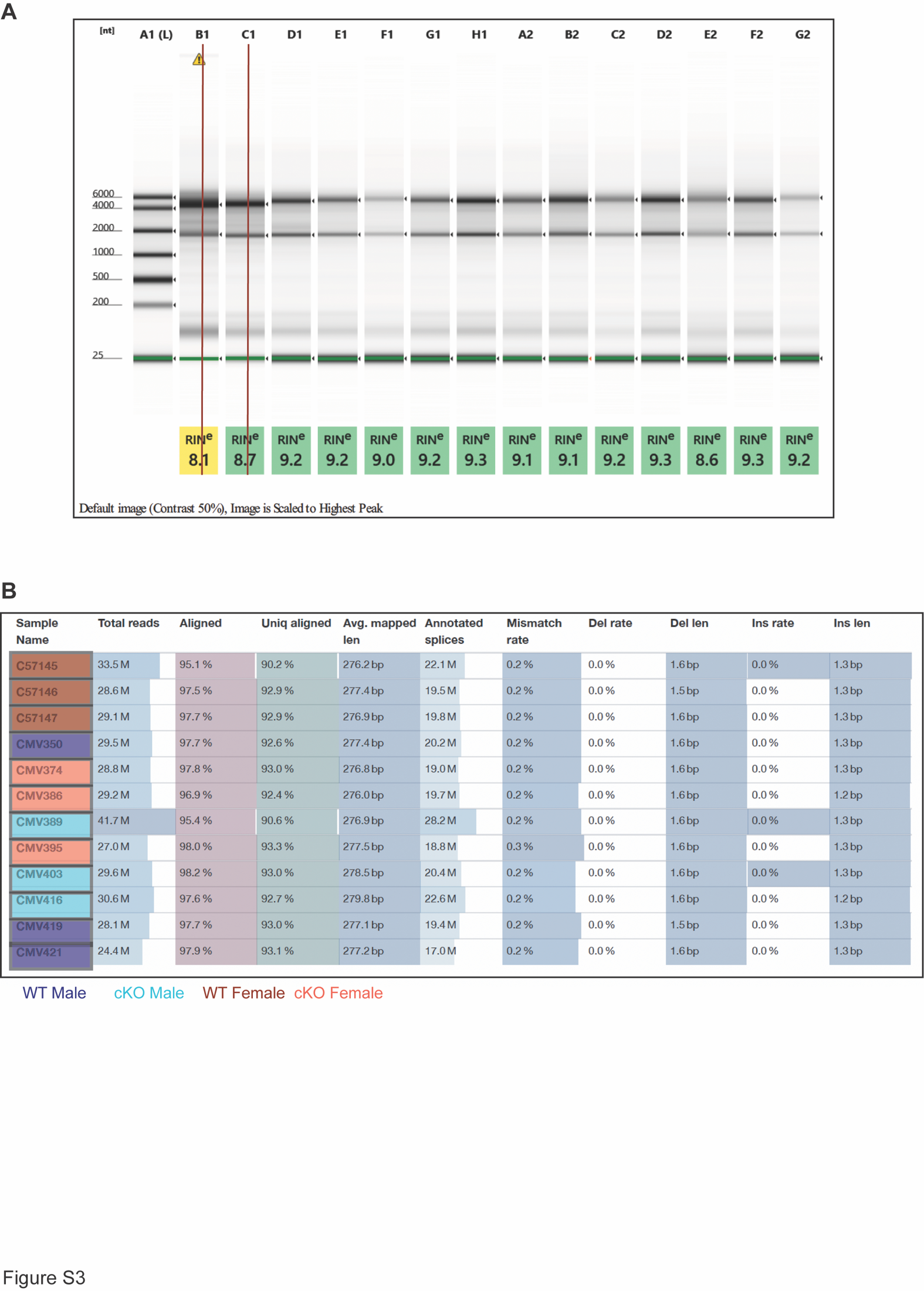


**Figure S3: RNA quality metrics for samples used in bulk RNA-seq** (**A**) Gel image and RNA Integrity Number (RIN) using RNA Tapestation. The first two columns (B1, C1, red lines) are samples that were not used in this manuscript. (**B**) A summary of data from STAR analysis of reads acquired from bulk RNA-seq. Data includes the total number of reads, read length, and the percent alignment.


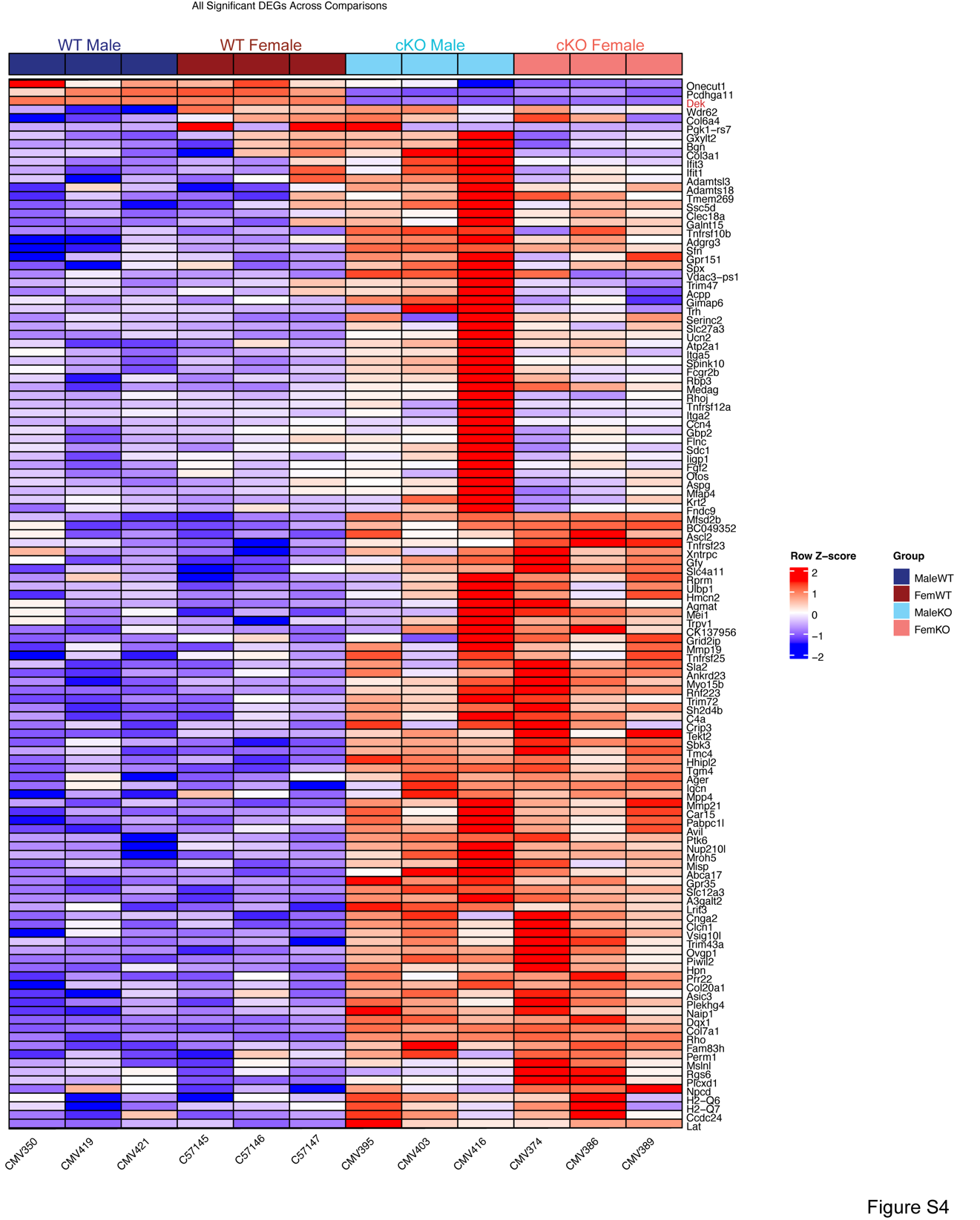


**Figure S4: Overall visualization of differentially expressed genes in *Dek* cKO mice*.*** A heatmap visualizes the Z-score of differentially expressed genes, using raw p value <0.05 and +/- 1.5-fold change as cutoffs.


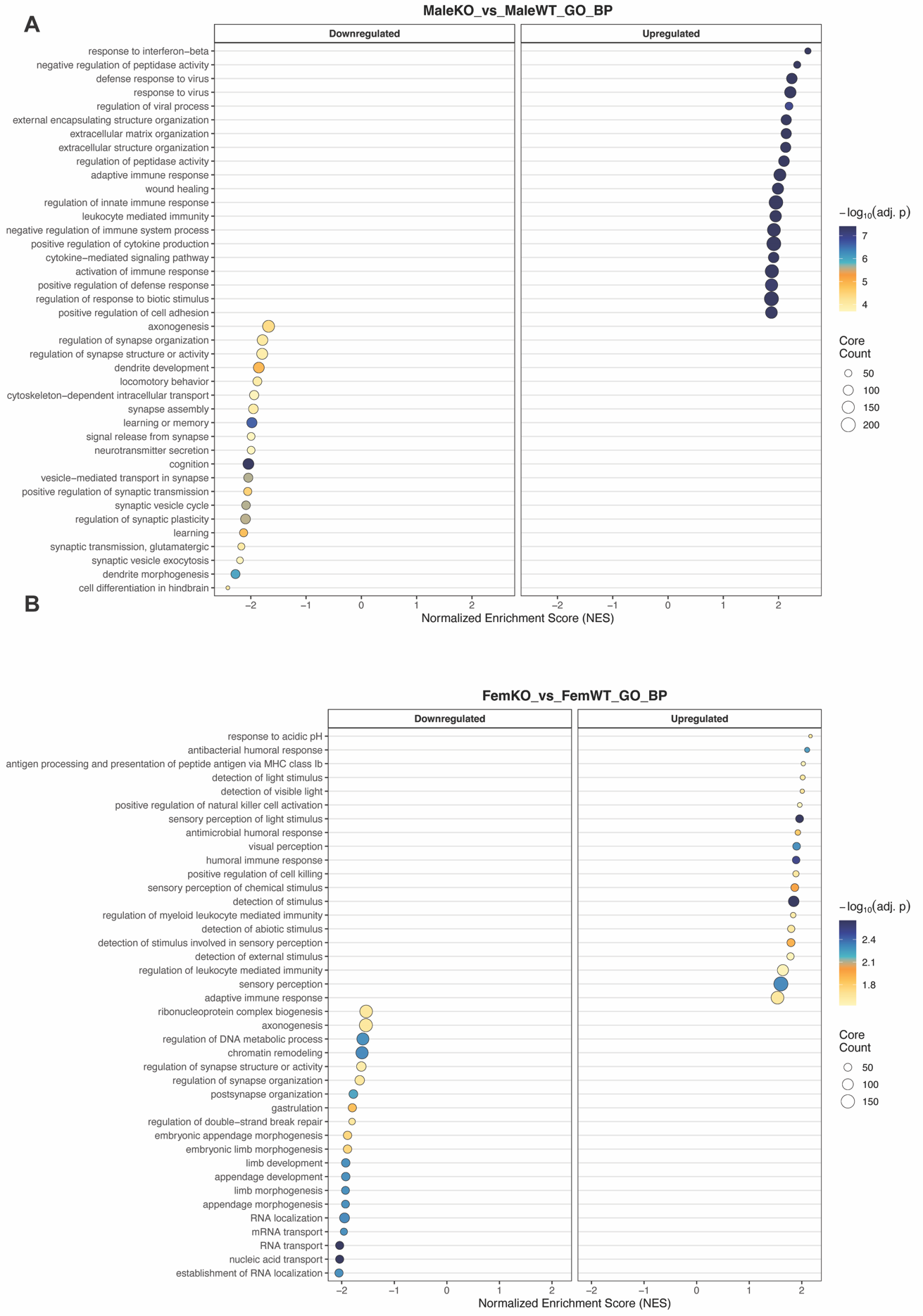


**Figure S5: GSEA evaluation for Biological Processes in male and female *Dek* cKO mice*.*** Gene Set Enrichment Analysis for Biological Processes for male (**A**) and female (**B**) *Dek* cKO hippocampal transcriptomes using DeSeq2-identified differential gene expression. Dot size represents the number of genes in the gene set differentially expressed in *Dek* cKO tissue vs WT tissue. The color scale represents p value, with darker blue indicating lower p values. The X axis graphs the gene set enrichment based on Normalized Enrichment Score (NES). Negative NES values represent down-regulation of a gene set and positive NES values depict up-regulation of a gene set. Data show that both males and females demonstrate up-regulation of inflammation and immune response processes and down-regulation of dendrite morphogenesis and synapse function.
